# Phase-based computational adaptive optics enables artifact-free super-resolution microscopy

**DOI:** 10.1038/s44172-026-00622-7

**Published:** 2026-03-09

**Authors:** Atsushi Matsuda, Carlos Mario Rodriguez-Reza, Yosuke Tamada, Yamato Matsuo, Takaharu G. Yamamoto, Takako Koujin, Peter M. Carlton

**Affiliations:** 1 Advanced ICT Research Institute, National Institute of Information and Communications Technology, Kobe, Japan; 2 Graduate School of Biostudies, Kyoto University, Kyoto, Japan; 3 School of Engineering, Utsunomiya University, Utsunomiya, Japan; 4 National Institute for Basic Biology, Okazaki, Japan; 5 Basic Biology Program, SOKENDAI, Okazaki, Japan; 6 Radiation Biology Center, Kyoto University, Yoshida-Konoecho, Kyoto, Japan

**Keywords:** Fluorescence imaging, Super-resolution microscopy, Adaptive optics, Wide-field fluorescence microscopy, Image processing

## Abstract

Adaptive optics has revolutionized biological microscopy by improving resolution and signal-to-noise ratio, yet its reliance on complex hardware and phototoxic wavefront sensing limits broader adoption. Here, we introduce ∅CAO, a computational phase-based adaptive optics technique that corrects optical aberrations in three-dimensional fluorescence microscopy without requiring specialized optics or training datasets. By leveraging phase transfer functions in the frequency domain, ∅CAO enables robust post-acquisition correction across diverse imaging modalities, including wide-field and structured illumination microscopy. Our method achieves substantial improvements in image fidelity, supports subregional aberration correction, and maintains performance under noisy conditions. Demonstrated on a range of biological specimens, including *Caenorhabditis elegans* and plant tissues, ∅CAO offers a scalable and accessible solution for high-resolution biological imaging, facilitating the broad deployment of adaptive optics approaches across the life sciences.

## Abstract (web summary)

Optical aberrations limit the resolution and reliability of many fluorescence microscopy techniques. Atsushi Matsuda and colleagues report a computational adaptive optics method that restores image quality and corrects aberration-related distortions without additional hardware.

## Introduction

The resolution of biological fluorescence microscopy has advanced significantly in recent years. Techniques such as structured illumination microscopy (SIM)^1–3^ and image scanning microscopy^4–6^, which utilize linear optics, can achieve up to twice the resolution of conventional microscopy. Even higher resolution is attainable through nonlinear optical methods, including stimulated emission depletion^7^ and single molecule localization microscopy^8,9^ or via expansion microscopy^10^, which physically enlarges the sample.

However, the increasing demand for higher resolution and deeper imaging has brought renewed attention to the challenge of optical aberrations, which arises from the complex refractive index variations within biological specimens. These aberrations distort both illumination and emission light paths, leading to image degradation. All forms of super-resolution microscopy are susceptible to such disturbances, resulting in reduced resolution and signal-to-noise ratio (SNR). In particular, SIM is prone to reconstruction artifacts caused by optical distortions, complicating image interpretation^11^.

To address sample-induced aberrations, adaptive optics (AO) has been integrated into microscopy^12^. AO systems measure the wavefront of light and compensate for aberrations using deformable mirrors or spatial light modulators. Classical Shack Hartmann wavefront sensors require point-like light sources, which are rare in biological samples. Moreover, the wavefront measured is localized and cannot be extrapolated to distant regions^13^. Consequently, AO systems must be adapted for biological imaging^12,14,15^. Sensor-less AO approaches introduce known aberrations via phase modulators and iteratively identify corrective phases across the field of view^14,16^. Both direct and indirect sensing methods have been successfully applied to super-resolution microscopy^17,18^. Despite their effectiveness, AO systems remain limited by their optical complexity, operational demands, and phototoxicity, restricting their use to specialized laboratories.

Several computational strategies have been developed to correct aberrations. Blind deconvolution^19–21^ simultaneously estimates the image and point spread function (PSF), has shown promise in restoring aberrated images^22^. However, it is an ill-posed, nonconvex optimization problem, particularly challenging in noisy conditions. Alternatively, deconvolution using measured or theoretical PSFs that incorporate aberrations can effectively restore image quality^11,23,24^, functioning as a computational analog to deformable mirrors to many degrees of freedom. Recent advances in deep learning-based AO have demonstrated successful aberration correction, but these methods typically require point sources or specialized training datasets^25–27^, limiting their generalizability across diverse biological samples.

In this study, we present a versatile computational method that emulates sensor-less AO as a post-processing step. Our approach, ∅CAO, operates on three-dimensional (3D) fluorescence images acquired via wide-field microscopy (WFM) and SIM without the need for prior training. ∅CAO effectively restores resolution and SNR in both synthetic samples and biological specimens, including multicellular nematode *Caenorhabditis elegans*.

## Results

### Phase as the dominant transfer function for optical aberrations in 3D frequency space

**Fig. 1:**
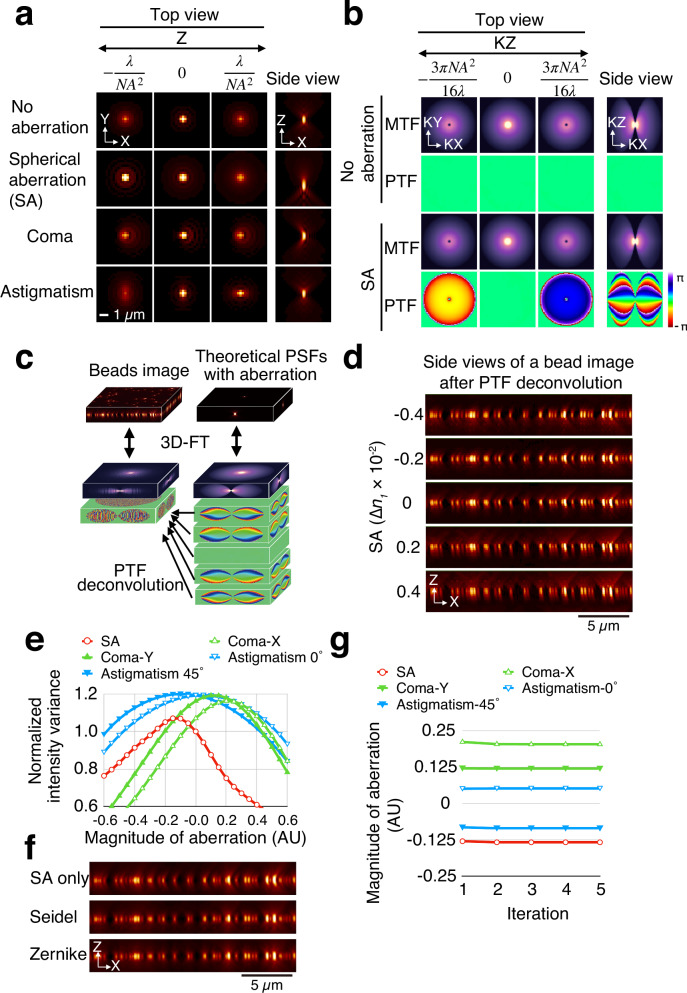
Phase modulation in the frequency domain of 3D images mimics optical phase modulation. **a** Theoretical 3D point spread functions (PSFs) with or without optical aberrations, shown in top and side views. Top views represent different focal planes. **b** 3D modulation transfer functions (MTFs) and phase transfer functions (PTFs) with and without spherical aberration (SA), shown in top and side views. Top views correspond to different axial frequencies. **c** A 3D image of fluorescent beads was deconvolved using a series of theoretical PTFs representing varying degrees of SA. Theoretical PTFs were derived from PSFs generated from corresponding wavefronts. “3D-FT” denotes 3D Fourier transformation. **d** Bead images modulated by PTFs with the indicated amounts of SA. Maximum intensity projections along the Y-axis are shown. **e** Plot of intensity variance from the images in **d** as a function of the introduced magnitude of aberration (arbitrary unit). **f** Bead images corrected for SA, Seidel aberrations (SA, coma, astigmatism) and Zernike aberrations of orders 1 to 3 (Wyant indices 4–24), including SA, coma, astigmatism, and trefoil/quatrefoil modes. **g** Measured aberration magnitude as a function of iteration number. The bead images and frequency space amplitude are displayed with gamma values of 0.5 and 0.4, respectively, to enhance visibility of low-intensity features.

We hypothesized that wavefront information is more readily discernible in 3D images—common in biological microscopy but rare in astronomy—than in 2D images. Optical aberrations are difficult to distinguish using a single 2D PSF, but they become more apparent in 3D PSFs (Fig. 1a). Theoretical PSFs of WFM without aberrations exhibit symmetry along both horizontal and vertical axes, whereas aberrated PSFs display asymmetry (Fig. 1a). This suggests that 3D image data may serve as a surrogate for wavefront sensing.

To computationally extract aberration information from 3D images, we analyzed their frequency domain representation. The Fourier transform of a PSF yields the optical transfer function (OTF), defined as $O\left({{\bf{k}}}\right)=A\left({{\bf{k}}}\right){{{\rm{e}}}}^{{{\rm{i}}}\phi \left({{\bf{k}}}\right)}$, where $A\left({{\bf{k}}}\right)$ is the amplitude (modulation transfer function, MTF) and $\phi \left({{\bf{k}}}\right)$ is the phase (phase transfer function, PTF). We compared theoretical MTFs and PTFs under conditions with or without optical aberrations and found that 3D PTFs were predominantly affected by aberrations, whereas changes in 3D MTFs were minimal (Fig. 1b, Supplementary Fig. 1). In contrast, 2D Fourier transforms revealed that 2D MTFs were more sensitive to aberrations than 2D PTFs (Supplementary Fig. 1). These findings indicate that 3D Fourier transformation preferentially transfers aberration information through the phase component, making PTF the primary carrier of optical distortion in 3D frequency space.

### Computational emulation of sensor-less adaptive optics via PTF deconvolution

Building on the observations described above, we developed a computational AO method using 3D images of 100-nm fluorescent beads acquired with WFM, which apparently contained spherical aberration (SA) and coma (Fig. 1c). For the given imaging conditions—including numerical aperture (NA) of the objective lens, refractive index of the immersion oil, pixel size, and emission wavelength—we computationally generated a series of pupil functions with varying degrees of positive and negative SA. These were used to simulate theoretical 3D PSFs containing SA. The PSFs were then Fourier transformed to obtain corresponding 3D MTFs and PTFs (Fig. 1c). The experimentally acquired bead images were deconvolved using only theoretical 3D PTFs. As anticipated, the PTFs effectively conveyed aberration information, allowing modulation of the SA content in the images (Fig. 1d). The axial intensity distribution became increasingly symmetric as amount of SA introduced by deconvolution approached the true aberration level (Supplementary Movie 1). When the refractive index mismatch (∆*n*_*1*_) was approximately −0.2 × 10^−2^, the axial intensity profile was maximally symmetric, with light converging at the bead center. At this point, the intensity variance—reflecting image sharpness—reached its peak (Fig. 1e “SA”, Supplementary Movie 1). Notably, total image intensity remained unchanged after PTF deconvolution, unlike conventional MTF-based deconvolution. Thus, image brightness can serve as a reliable metric for evaluating aberration correction. The ∆*n*_*1*_ value that maximized intensity variance (approximately −0.125 × 10^−2^) corresponded to the actual SA present in the image.

At this stage, the bead image was symmetric along the optical axis but remained asymmetric along the horizontal axis due to residual aberrations (Fig. 1f, “SA only”). We repeated the same procedure for other Seidel-type aberrations, including coma and astigmatism, following the process analogous to sensor-less AO (Supplementary Fig. 2). The intensity variance for other Seidel aberrations—including spherical aberration (SA), coma, and astigmatism—also exhibited simple convex profiles (Fig. 1e). Convergence was achieved within a single measurement cycle, and additional iterations did not substantially alter the result for this sample (Fig. 1g). Finally, we applied a composite correction using the sum of all Seidel aberration types (Fig. 1f “Seidel”). Additionally, incorporating higher-order Zernike polynomials further improved image symmetry beyond that achieved by correcting only the three primary Seidel aberrations (Fig. 1f “Zernike”).

Importantly, wavefront measurements were independent of the underlying object structure. We successfully extracted wavefront information from biological specimens such as actin filaments in cultured cells (Supplementary Fig. 3) or cell nuclei (see below).

We refer this computational AO approach as *phi C*omputational *A*daptive *O*ptics (∅CAO), as it relies on phase (∅) information in the frequency domain.

### ∅CAO reliably estimates aberration parameters from images with complex structures and noise

Computational approaches are often perceived as less effective than their optical counterparts. However, the following data demonstrate that ∅CAO performs comparably to optical methods in many aspects—and even exceeds them in certain scenarios.

**Fig. 2:**
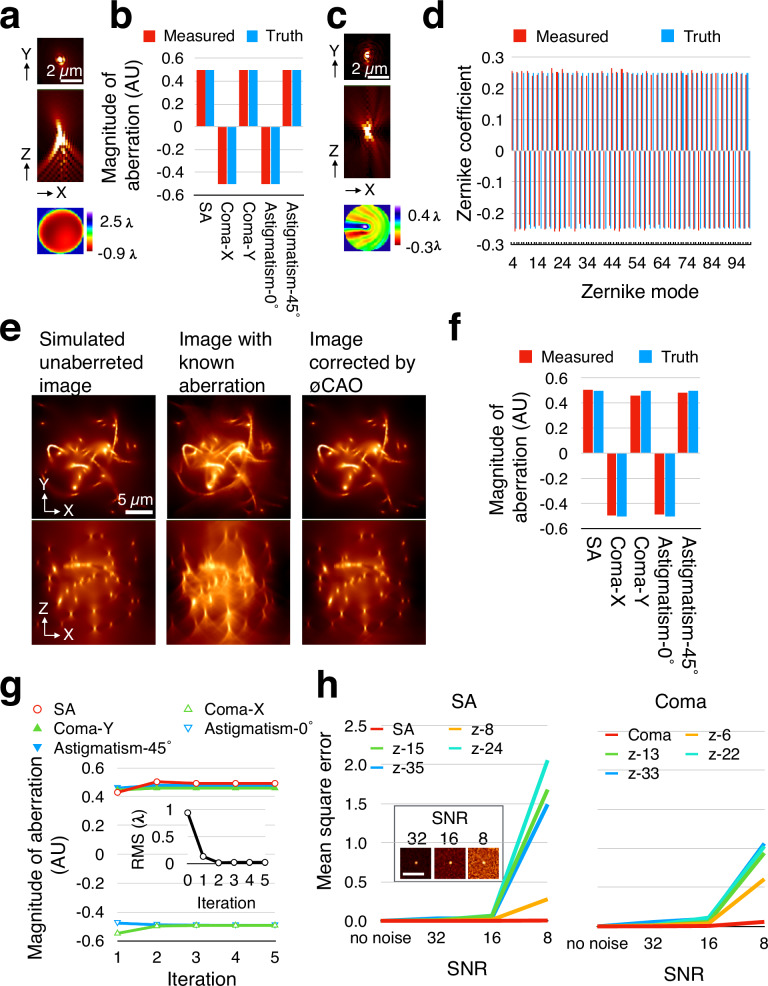
∅CAO accurately estimates aberrations in simulated images under noisy conditions. Theoretical 3D point objects with large Seidel (**a**) or Zernike (**c**) aberrations and corresponding wavefronts. Point objects were displayed with a gamma = 0.5 to enhance low-intensity features. **b**, **d** Measured versus ground-truth aberration magnitudes estimated by ∅CAO from images in **a**, **c**. Zernike mode are numbered according to Wyant indices. Values shown correspond to the 2nd and 20th iterations for **b**, **d**, respectively. **e** Top and side views of simulated filament-like structures: unaberrated, aberrated, and aberrated but corrected by ∅CAO. **f** Measured versus ground-truth aberration magnitudes from **e**, shown after the 2nd iterations. **g** Aberration magnitude estimated by ∅CAO across five iterations. Inset shows root mean square (RMS) wavefront error; zero iteration indicates no correction. **h** Simulation results illustrating measurement accuracy for spherical aberration (SA), coma and corresponding Zernike modes (denoted as “z-“ followed by Wyant indices) under varying signal-to-noise ratios (SNRs). Normalized mean square error of the measured aberration values is plotted. Insets show top views of simulated single-point light source images used in the analysis.

Firstly, we measured known magnitudes of Seidel aberrations in point objects generated by computer simulations (Fig. 2a). ∅CAO accurately estimated the ground-truth values (Fig. 2b). While small aberrations did not require iteration, larger aberrations needed two to four iterations to achieve accurate estimation (Supplementary Fig. 4a). Similarly, ∅CAO correctly estimated a complex combination of 96 Zernike modes (Fig. 2c, d). For Zernike modes, iteration was necessary not only for larger coefficients but also for cases involving multiple modes (Supplementary Fig. 4b–f).

Secondly, we tested a realistic simulated image of a filament-like structure for aberration measurement. Again, ∅CAO successfully estimated the ground-truth aberrations (Fig. 2e, f). Acceptable results were obtained without iteration, though more than two iterations were required for precise estimation (Fig. 2g). Comparable results were observed for more complex Zernike modes (Supplementary Fig. 5).

Thirdly, we evaluated the noise tolerance of ∅CAO through computer simulations. As expected, the mean square error relative to the ground truth increased as the SNR decreased (Fig. 2h). At a low peak SNR of 8, higher-order Zernike modes exhibited noticeable deviations from the true values (Fig. 2h, Supplementary Fig. 6). Nevertheless, Seidel aberrations were accurately estimated even under these noisy conditions (Fig. 2h, Supplementary Figs. 6). These findings suggest that ∅CAO offers noise tolerance at least an order of magnitude greater than conventional Shack-Hartmann wavefront sensors, and that higher-order aberrations can be reliably measured when SNR exceeds 16.

We further investigated whether ∅CAO could resolve closely spaced point sources that appeared fused due to aberrations. Simulated two-point light sources positioned along the optical axis were initially indistinguishable (Supplementary Fig. 7). After aberration correction with ∅CAO, the two sources were fully resolved (Supplementary Fig. 7, right). The intensity profile revealed a Strehl ratio of 1.0, indicating complete recovery of both resolution and brightness (Supplementary Fig. 7, bottom). Although SNR improvement in noisy conditions involves multiple factors, ∅CAO achieved an approximately 1.5–2.5 fold enhancement in SNR compared to the original image (Supplementary Fig. 8, Supplementary Note 1).

Another factor influencing ∅CAO performance is the number of optical sections acquired. Deconvolution cannot recover intensity distributions if photons are not captured. We simulated intensity recovery as a function of section number and SA magnitude. Even with substantial SA (∆*n*_*1*_ = 0.01; Supplementary Fig. 9a), 64% and 78% of peak intensity were recovered from 3 and 5 optical sections, respectively, at Nyquist sampling frequency (Supplementary Fig. 9b). In most cases, 90–100% of peak intensity was restored (Supplementary Fig. 9b).

### ∅CAO recovers resolution in experimental imaging data

**Fig. 3:**
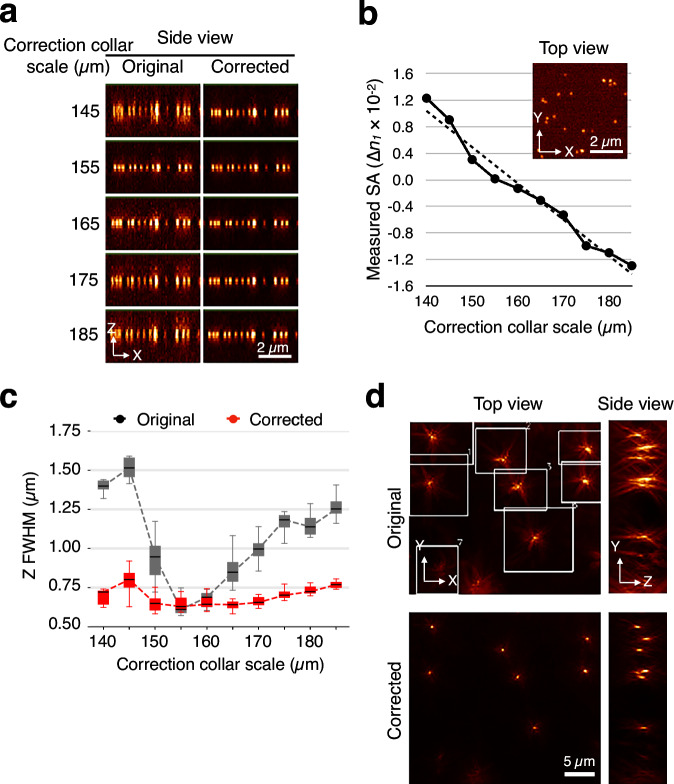
∅CAO accurately estimates aberrations in real samples and restores resolution, even in highly distorted images. **a** Side views of yellow-green 100-nm fluorescent bead images acquired under varying correction collar positions of the objective lens, along with the same images corrected using ∅CAO for spherical aberration (SA). **b** Plot of the measured SA values from the bead images in **a** as a function of correction collar scale. The inset shows a top view of the bead image acquired at a collar setting of 185 µm. **c** Box plots of axial full width at half maximum (FWHM) of the bead images in **a**, plotted against correction collar scale (*n* ≥ 10). **d** Highly aberrated beads images observed through plant tissue were corrected using ∅CAO using 96 Zernike modes and subregional aberration measurement. Maximum intensity projections are shown in top and side views. Subregions used for aberration measurement are indicated by white boxes in the original image.

Having tested with simulated data, next, we assessed measurement accuracy using real samples of 100-nm fluorescent beads acquired under varying correction collar positions of the objective lens and different immersion oil refractive indices to introduce known levels of SA (Fig. 3a, Supplementary Fig. 10). The image obtained at a correction collar setting of 155 µm exhibited minimal aberration, while deviations above or below this value introduced SA. After applying ∅CAO, all images were restored to a state equivalent to the aberration-free condition (Fig. 3a, Supplementary Fig. 10a, d). The measured SA values showed a linear correlation with the correction collar scale, confirming the reproducibility of the method (Fig. 3b, Supplementary Fig. 10e). In contrast, measured coma and astigmatism remained largely unchanged across conditions (Supplementary Fig. 10b, f). The full width at half maximums (FWHMs) of the bead images was consistently corrected to match the FWHM observed at the optimal collar setting of 155 µm (Fig. 3c, Supplementary Fig. 10c).

Fluorescent beads imaged through *Physcomitrella patens* leaf cells appeared highly distorted, with spatially variable aberrations^28^. We investigated whether ∅CAO could correct such severe distortions. A key advantage of computational methods over optical approaches is the ease of implementing regional corrections. By performing sub-regional aberration measurements using 96 Zernike modes and leveraging the high SNR of the fluorescent beads, ∅CAO successfully corrected spatially variant and complex aberrations across the entire field of view (Fig. 3d, Supplementary Fig. 11). Even more severely distorted bead images were restored using 10 iterative corrections with 96 Zernike modes (Supplementary Fig. 12). Such extensive correction—across multiple sub-regions and using numerous Zernike modes—is challenging for traditional sensor-less AO systems, but readily achievable with computational methods.

### ∅CAO enhances biological imaging in WFM

**Fig. 4:**
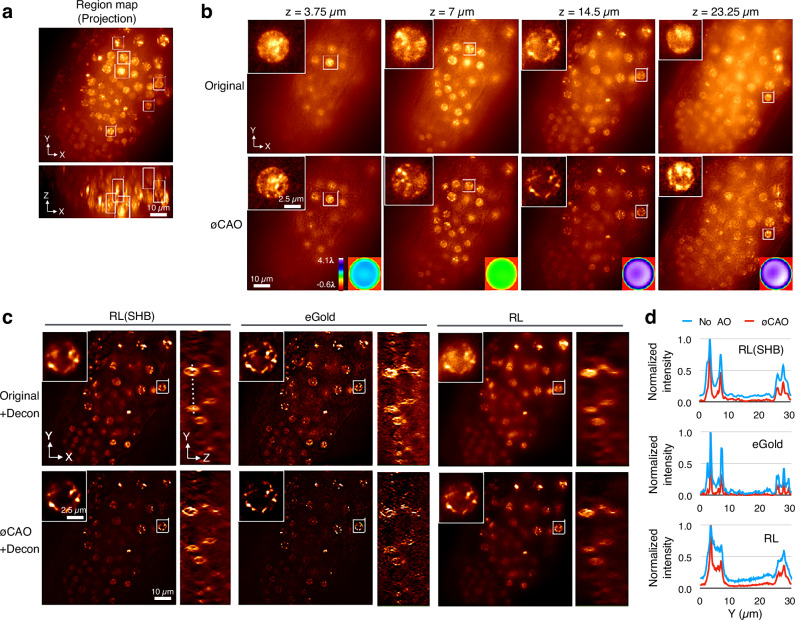
∅CAO enhances resolution and signal-to-noise ratio (SNR) in wide-field microscopy of live *C. elegans* embryos. Histone H2B-GFP was expressed in live *C. elegans* embryos to visualize nuclear chromatin. **a** Boxed regions indicate areas where optical aberrations were measured. Images are shown as maximum intensity projections along the Z- and Y-axes. **b** Representative single optical sections of the original and corrected images following Seidel aberration correction. Axial positions at the top denote the distance from the bottom of the animal. **c** Representative optical sections of the original and ∅CAO-corrected images after deconvolution using three methods: “RL(SHB)”, Richardson-Lucy with Scaled Heavy Ball algorithm^47^; “eGold”, enhanced Gold algorithm^48^; “RL”, conventional Richardson-Lucy algorithm. **d** Line profiles of original and ∅CAO-corrected images derived from the dotted line shown in the side view of the original image deconvolved with “RL(SHB)”. Scale bars: 10 µm for the full-field view in **a**–**c**; 2.5 µm for the inset in **b**, **c** showing the aberration measurement regions.

We applied ∅CAO to WFM images of live *C. elegans* embryos expressing GFP-tagged histone H2B. Seidel aberrations were measured in six localized regions and corrections were applied to adjacent areas. In all corrected regions, condensed chromosome structures became clearly visible due to improved resolution and SNR (Fig. 4a, b). Deconvolution using an unaberrated PSF was more effective after aberration removal, as confirmed by three different algorithms (Fig. 4c). In all case, deconvolution performance improved in the ∅CAO-processed image (Fig. 4c, d).

We also tested an image previously used in other deconvolution studies (Supplementary Fig. 13). Again, ∅CAO enhanced deconvolution efficiency, confirming accurate aberration estimation. These results demonstrate that ∅CAO enables sub-regional aberration measurement and correction from a single image acquisition of biological samples.

Sub-regional analysis also reduces computational time for wavefront estimation. Using a pupil size of 256 × 256 pixels, the Python program required only 5.2 ± 0.60 s (mean ± standard deviation) to measure aberrations in a single region (average size: 49 × 89 × 87 pixels) without iteration. Correction requires 143 ± 0.2 s to process the entire image (196 × 1024 × 1024 pixels) across six sub-regions on an Apple M3 processor (ARM64E, 3.57 GHz) using a single processing thread.

∅CAO can also be applied following correction of sample drift, which frequently occurs during time-lapse imaging of motile specimens such as live worms. After computational drift correction, regional aberrations were successfully eliminated using ∅CAO (Supplementary Fig. 14). Such operations are challenging to implement optically, but readily achievable through computational methods.

### ∅CAO enhances 3D-SIM by correcting optical aberrations

AO is critical for achieving high-resolution imaging in super-resolution microscopy. In 3D-SIM, optical aberrations not only degrade resolution and SNR but also introduce reconstruction artifacts. Given that 3D-SIM builds upon WFM, ∅CAO is well-suited to enhance 3D-SIM images.

**Fig. 5:**
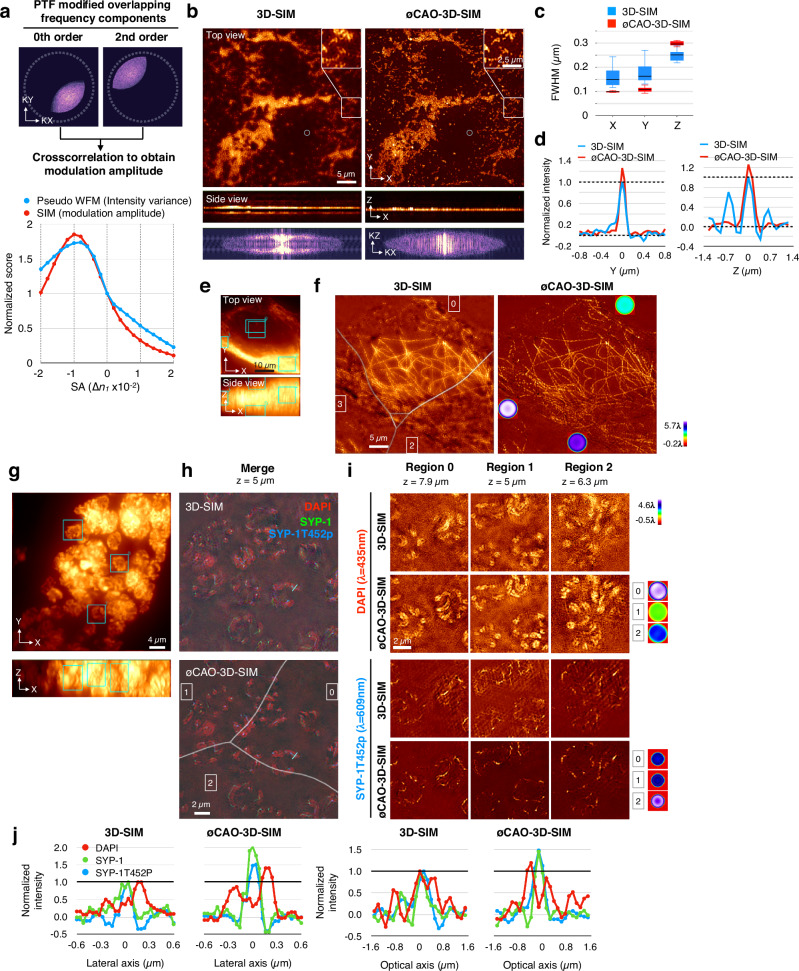
∅CAO restores optimal resolution in 3D structured illumination microscopy (SIM). **a** Selected regions of the frequency distribution used to measure modulation amplitudes are shown at the top displayed with a gamma value of 0.4. A plot of image quality metrics derived from these measurements is shown below. The pseudo wide-field microscopy (WFM) method evaluates intensity variance, whereas the SIM method uses modulation amplitude. **b** Original and ∅CAO-corrected reconstructed images of yellow-green 100-nm beads using Seidel aberration correction. Middle panel show side views as maximum intensity projections along the Y-axis; bottom panel show corresponding frequency distributions at zero frequency along the Y-axis, displayed with a gamma value of 0.4. **c** Box plots showing full width at half maximum (FWHM) (µm) of 100-nm beads (*n* = 13) before and after correction. **d** Intensity profiles along the Y- and Z-axes of the beads indicated by the circle in (**b**). **e** Regions used for aberration measurement in a fixed HeLa cell stained for microtubules. Maximum intensity projections along the Z- and Y-axes are shown. **f** 3D-SIM reconstruction of microtubules in a HeLa cell before and after ∅CAO correction using Seidel aberrations. Boundaries between subregions (numbered 0, 2, and 3) to apply correction are indicated by gray lines in the uncorrected image. Corresponding wavefronts used for correction are shown in the corrected image. **g** Maximum intensity projection along the Z- and Y-axes of DAPI-stained chromosomes in meiotic prophase of fixed *C. elegans* embryos. Subregions used for aberration measurement are indicated by blue boxes. **h** Multicolor optical section of 3D-SIM with or without ∅CAO correction using Seidel aberrations. White lines in the merged image indicate boundaries between subregions (numbered 0, 1, and 2) corrected using distinct wavefronts. Corresponding wavefronts are shown in the corrected images. Pseudo-color was applied to the merged image. **i** Reconstructed 3D-SIM images in unoptimized blue (435 nm) and red (609 nm) channels. Representative images from three subregions (indicated in **h**) are shown. Wavefronts used for correction are displayed on the right. **j** Line profiles along the lateral or axial directions of the blue line in the merged images in **h**, comparing images with or without ∅CAO correction.

To measure aberrations, pseudo-WFM images—generated by summing all phases of raw 3D-SIM data—offer a high SNR but lack the high-resolution information intrinsic to SIM. Conversely, raw 3D stacks individual illumination phases retain high-resolution content but suffer from low SNR. To leverage both, we employed a modulation amplitude-based measurement during the 3D-SIM reconstruction process, which adjusts line spacing, angle, amplitude, and phase for each frequency component^2,29^. The modulation amplitude was quantified via cross-correlation of the overlapping frequency components, multiplied by the OTFs of the interference orders (Fig. 5a). By varying only the PTF, we iteratively identified the aberration level that maximized modulation amplitude (Fig. 5a). This information was then used to generate theoretical 3D-SIM PSFs for deconvolution.

The 3D-SIM images of 100-nm beads and microtubules in fixed HeLa cells, acquired with mismatched immersion oil to introduce SAs, exhibited reduced resolution and artifacts such as hatching, and ghosting^11^ (Fig. 5b, f). Application of ∅CAO effectively removed these distortions, yielding images comparable to those obtained under ideal conditions (Fig. 5b, f). Axial frequency distributions, initially irregular, became more uniform post-correction (Fig. 5b). Lateral FWHM improved by ~1.63 + 0.36 fold (*n* = 13), reaching the expected ~100 nm resolution (Fig. 5c). Intensity profiles showed reduced asymmetry and increased peak intensity (1.30 ± 0.15-fold, Fig. 5d). Although axial FWHM slightly increased from 251 to 294 nm (Fig. 5c), it approached the expected value (~290 nm) under aberration-free conditions. The overly sharp axial FWHM observed without ∅CAO likely resulted from negative values between multiple axial peaks (Fig. 5d), reflecting abnormal amplitude distribution in frequency space (Fig. 5b). Microtubule images were restored with high contrast and fine filament detail, and correction boundaries were nearly imperceptible, indicating precise regional aberration estimation (Fig. 5f).

Multicolor 3D-SIM is particularly susceptible to SAs due to wavelength-dependent refractive index variations. Typically, only one channel is optimally corrected, while others suffer from optical degradation. We applied ∅CAO to multicolor 3D-SIM images of *C. elegans* meiotic chromosomes (Fig. 5g). In this case, optics were optimized for the green channel (center emission wavelength, 528 nm), resulting in pronounced artifacts in the blue (435 nm) and red channels (609 nm). These included shadows and honeycomb patterns that obscured structural interpretation (Fig. 5h, i). ∅CAO successfully restored image quality across all channels, revealing paired homologous chromosomes with a ~400 nm central gap in the blue channel (4’,6-diamidino-2-phenylindole [DAPI] staining), and synaptonemal complex proteins (SYP-1 and its phosphorylated form SYP-1T452p) localized within the gap (Fig. 5i, j, Supplementary Fig. 15). Peak intensity increased by 1.4–2.0-fold depending on the channel, while noise levels were reduced (Fig. 5i, j). These results confirm that ∅CAO effectively enhance 3D-SIM imaging and restores super-resolution across multiple wavelengths.

## Discussion

In this study, we demonstrated that the procedures for optical sensor-less AO can be faithfully reproduced through computational means. Our method, ∅CAO, operates without the need for additional hardware and can be applied post-acquisition, thereby enhancing the effectivity of data collection. It does not rely on training datasets or point-like light sources, and it is capable of restoring aberrated optical paths to near-ideal conditions—even in noisy images (Figs. 2–5). Moreover, we showed that regional aberration correction is significantly easier to implement after image acquisition over a large field of view. Computational time can be further improved by optimizing the codebase with faster programming languages and parallel processing. Importantly, ∅CAO can be immediately deployed on commercial microscopes without any hardware modifications. These advantages highlight ∅CAO as a practical alternative to conventional AO, which often requires intricate optical setups, expert operation, and carries risks of phototoxicity.

Because ∅CAO relies on the generation of theoretical PSFs, its implementation must be tailored to specific microscopy systems. We successfully generated aberration-inclusive theoretical PSFs for WFM and 3D-SIM and applied ∅CAO to correct their images. In particular, ∅CAO effectively eliminated common artifacts of 3D-SIM and restored optimal resolution (Fig. 5). Although the current implementation does not modify the illumination optics, the large beam size in 3D-SIM renders it relatively insensitive to moderate aberrations. Furthermore, ∅CAO is well-suited for expansion microscopy^30,31^, where enlarged samples often suffer from increased SA due to their size. In combination with WFM and 3D-SIM, ∅CAO can improve expansion microscopy images without altering the imaging protocol. Future advancements will enable ∅CAO to be applied across a broader range of microscopy modalities, such as confocal, light-sheet, two-photon, and advanced super-resolution techniques.

Because ∅CAO was originally developed to enhance 3D-SIM, our test images were limited to moderately sized specimens, such as nematodes. Nevertheless, we confirmed that correcting complex aberrations is not inherently limited, provided the image has sufficient SNR to resolve higher-order Zernike modes (see Fig. 2h, Supplementary Fig. 6). Indeed, we demonstrated accurate restoration of severely aberrated simulated images and bead images acquired through plant tissue (see Figs. 2a–g, 3d; Supplementary Figs. 4, 5, 11, and 12). Thus, in theory, ∅CAO should be applicable to more challenging biological scenarios, such as live imaging of neural activity in the mouse brain.

While ∅CAO shares similarities with blind deconvolution, it offers a key advantage. Traditional blind deconvolution requires solving for three unknowns—MTF, PTF, and the true image—making it a highly ill-posed problem in 3D. In contrast, ∅CAO solves only for the PTF and the true image, reducing the task to a convex optimization that reliably converges (see Fig. 1g, Supplementary Fig. S4). Future developments in computational AO, including deep learning, should incorporate PTF as a central component. However, ∅CAO may be less photon-efficient because it ignores MTF information. Further comparison studies are needed across computational approaches.

Although ∅CAO is robust like sensor-less AO, it shares a common limitation at image boundary. Unlike optical methods, computational AO relies solely on information within the image. If an object is truncated at the boundary, optical theory breaks down locally, potentially leading to incorrect results. Therefore, careful region selection is essential. Integrating AI-based automated region selection for aberration measurement could enable fully automated ∅CAO workflows, improving both speed and accuracy.

Recent advances have introduced new computational methods for measuring optical aberrations. Light-field microscopy corrects aberration by shifting the images acquired from different angles, avoiding the SNR loss typical of deconvolution^15,32^. However, this approach requires a microlens array, which increases optical complexity compared to purely computational adaptive optics. The latest deep-learning-based methods employ self-supervised schemes^33^ or leverage Fourier-domain information^34^ eliminating the need for sample-specific training. While promising, these methods do not provide a theoretical basis for wavefront measurement. Furthermore, to our knowledge, no computational AO method has yet been applied to 3D-SIM.

∅CAO is well suited to live, time-lapse imaging applications. Although real-time correction remains challenging due to computation times, ∅CAO enables regional correction which is difficult to achieve optically. Its post-processing approach is advantageous for multi-point time-lapse imaging. Moreover, ∅CAO could be extended for real-time aberration correction by integrating deformable mirrors or spatial light modulators, as wavefront sensing can be computed within seconds. This capability would make ∅CAO suitable for dynamic imaging and potentially useful in routine medical image analysis. To further expand its utility, computational AO methods such as ∅CAO and blind deconvolution should be enhanced through hardware integration, AI-driven object recognition, and complementary technologies such as deep learning, phase diversity and phase retrieval^35–37^.

## Methods

### Creation of wavefronts

We generated the defocus and SA wavefronts using the method originally described by Gibson and Lanni^38,39^. Defocus was introduced by varying the thickness of the immersion medium between the coverslip and the objective lens. SA wavefronts were calculated as the optical path difference between ideal and nonideal light trajectories. Although SA can be introduced by altering the sample’s refractive index, this also depends on the object’s distance from the coverslip. To reduce parameter complexity, we introduced SA by modifying the refractive index of the immersion medium.

Comatic aberration wavefronts were defined as:

1 $${W}_{c}\left(u,v,{\alpha }_{u},{\alpha }_{v}\right)={\alpha }_{u}\left({u}^{2}+{v}^{2}\right)u+{\alpha }_{v}\left({u}^{2}+{v}^{2}\right)$$

where ${\alpha }_{u}$ and ${\alpha }_{v}$ are the coma coefficients along the $u$ and $v$ axes, respectively.

Astigmatism wavefronts were defined as

2 $${W}_{g}\left(u,v,{\alpha }_{g}\right)={0.5\cdot \alpha }_{g}{u}^{2}$$

where ${\alpha }_{g}$ is the astigmatism coefficient along the $u$ axis. Orthogonal astigmatism was generated by rotating the $(u,v)$ coordinate system by 45°.

Zernike aberrations were constructed using standard Zernike polynomials.

The tilt wavefronts were defined as:

3 $${W}_{t}\left(u,v,x,y\right)=0.5\cdot \lambda \left(-x\cdot u-y\cdot v\right)$$

where $x$ and $y$ represent lateral shifts along the X and Y axes, respectively.

### Creation of the PSF and OTF

The pupil function is defined as follows:

4 $$P\, \left(u,v,{NA},\lambda \right)=\left\{\begin{array}{cc}1, & {u}^{2}+{v}^{2} < {\left(\frac{{NA}}{\lambda }\right)}^{2}\\ 0, & {\mbox{otherwise}}\end{array}\right.$$

To incorporate aberrations, we modified the pupil function by summing wavefront components:

5 $${P}^{{\prime} }\left(u,v,z,\psi ,\alpha \right)=P\, \left(u,v\right){{{\rm{e}}}}^{{{\rm{i}}}\frac{2{{\rm{\pi }}}}{\lambda }\left\{\mathop{\sum }\limits_{\psi =1}^{n\psi }\left[{W}_{a}\left(u,v,\psi ,\alpha \right)+{W}_{d}\left(u,v,z\right)+{W}_{t}\left(u,v,\psi ,\alpha \right)\right]\right\}}$$

where

- ${W}_{\!a}$ is the aberration wavefront for types ($\psi$) and magnitude ($\alpha$),
- ${W}_{\!d}$ is the defocus wavefront at axial position $z$,
- ${W}_{\!t}$ is the tilt wavefront,
- $n\psi$ is the number of aberration type.

Because the original Gibson and Lanni^38^ uses a 1D Bessel function, which is not easily applicable to a 2D wavefront, we adopted the method by Hanser et al.^37^ to compute theoretical PSFs via 2D inverse Fourier transforms:

6 $${h}^{{\prime} }\left(x,y,z,\psi ,\alpha \right)={{{\mathscr{F}}}}_{2D}^{-1}\left[{P}^{{\prime} }\right]\cdot {{{{\mathscr{F}}}}_{2D}^{-1}\left[{P}^{{\prime} }\right]}^{* }$$

3D PSF was obtained by stacking 2D slices at sequential defocus positions. After transformation, the central region of the PSF was cropped to match the desired resolution.

A 3D OTF was obtained by applying a 3D Fourier transform to the 3D PSF

7 $${O}^{{\prime} }\left({k}_{x},{k}_{y},{k}_{z},\psi ,\alpha \right)={{{\mathscr{F}}}}_{3D}\left[{h}^{{\prime} }\right]$$

From this, the MTF and a PTF were extracted as:

8 $${{O}^{{\prime} }=A}^{{\prime} }\left({k}_{x},{k}_{y},{k}_{z},\psi ,\alpha \right){{{\rm{e}}}}^{{{\rm{i}}}{{{ \varnothing }}}^{{\prime} }\left({k}_{x},{k}_{y},{k}_{z},\psi ,\alpha \right)}$$

where ${A}^{{\prime} }$ and ${ \varnothing }^{{\prime} }$ represent the amplitude and phase components of the aberrated OTF, respectively.

### Wavefront measurement from 3D WFM images

To measure aberration from 3D WFM images, we first calibrated the vertical and lateral shifts induced by each aberration type. These shifts were then computationally canceled by adjusting the corresponding defocus and tilt wavefronts. Using these corrected parameters, we generated theoretical pupil functions, PSFs, OTFs, and PTFs.

The acquired 3D image stack $d\left(x,y,z\right)$ was transformed into frequency space via a 3D Fourier transform:

9 $$D\left({k}_{x},{k}_{y},{k}_{z}\right)={{{\mathscr{F}}}}_{3D}\left[d\left(x,y,z\right)\right]$$

To simulated phase-only deconvolution, we applied the following operation:

10 $${D}^{{\prime} }\left({k}_{x},{k}_{y},{k}_{z},\psi ,\alpha \right)=\left\{\begin{array}{ll}\frac{D\left({k}_{x},{k}_{y},{k}_{z}\right)}{{A\left({k}_{x},{k}_{y},{k}_{z}\right){{\rm{e}}}}^{{{\rm{i}}}{{{ \O }}}^{{\prime} }\left({k}_{x},{k}_{y},{k}_{z},\psi ,\alpha \right)}+{w}^{2}}, & \left({k}_{x} < {k}_{{xmax}},{k}_{y} < {k}_{{ymax}},{k}_{z} < {k}_{{zmax}}\right)\\ 0, \hfill & \left({k}_{x}\ge {k}_{{xmax}},{k}_{y}\ge {k}_{{ymax}},{k}_{z}\ge {k}_{{zmax}}\right)\end{array}\right.$$

where:

- $A$ is the theoretical MTF without aberrations,
- ${ \varnothing }^{{\prime} }$ is the phase component of the aberrated OTF,
- $w$ is the Wiener filter constant (set to 0.22 in this study),
- ${k}_{{xmax}}$, ${k}_{{ymax}}$, ${k}_{{zmax}}$ are the maximum frequencies of the imaging system along ${k}_{x}$, ${k}_{y}$ and ${k}_{z}$ axes, respectively.

The corrected image was obtained by inverse 3D Fourier transformation:

11 $${d}^{{\prime} }\left(x,y,z,\psi ,\alpha \right)={{{\mathscr{F}}}}_{3D}^{-1}\left[{D}^{{\prime} }\right]$$

To evaluate image quality, we computed the variance of the squared intensity:

12 $$v\left(\psi ,\alpha \right)={{\mbox{Var}}}\left\{{{\mathbb{R}}}_{\ge 0}{\left[{d}^{{\prime} }\left(x,y,z,\psi ,\alpha \right)\right]}^{2}\right\}-{{\rm{\gamma }}}{{\rm{Var}}}\left\{{{\mathbb{R}}}_{ < 0}{\left[{d}^{{\prime} }\left(x,y,z,\psi ,\alpha \right)\right]}^{2}\right\}$$

where:

- ${{\mathbb{R}}}_{\ge 0}$ and ${{\mathbb{R}}}_{ < 0}$ are set of real numbers greater or less than 0, respectively
- ${{\rm{\gamma }}}$ is a penalty parameter for the negative intensity (set to 10).

Prior to variance calculation, the image spectrum was filtered using a theoretical 3D bandpass filter^40^ defined as:

13 $$C\left({k}_{x},{k}_{y},{k}_{z},\chi ,\lambda ,\theta \right)=\left\{\begin{array}{cc}1, & 2\cdot \frac{2{{\rm{\pi }}}}{\chi \lambda }\left(|{k}_{l}|\sin \theta -|{k}_{s}|\cos \theta \right)\ge \sqrt{{k}_{l}^{2}+{k}_{s}^{2}}\\ 0, & {\mbox{otherwise}}\end{array}\right.$$

where:

- $\chi$ is a scaling factor (set to 1.8),
- ${k}_{l}$ and ${k}_{s}$ are lateral and axial frequency coordinates,
- $\theta =\sin \frac{{NA}}{n}$ is the collection angle.

The optimal aberration magnitude $\varepsilon \left(\psi \right)$ was determined by maximizing the variance:

14 $$\varepsilon \left(\psi \right)=\mathop{{{\rm{argmax}}}}\limits_{\alpha }\left[v\left(\psi ,\alpha \right)\right]$$

To determine $\varepsilon (\psi )$, we first computed $v\left(\psi ,\alpha \right)$ at five initial sampling points of $\alpha$(Supplementary Fig. 16). The initial sampling step was ∆*n*_*1*_ = 0.01 for SA, $\alpha$=0.125 for coma and astigmatism and $\alpha$=0.5 for Zernike coefficients. Additional points (typically two) were then added to refine the sampling range (Supplementary Fig. 16a). Finally, a fifth-order polynomial fit was applied to five points near the peak to estimate $\varepsilon (\psi )$ precisely (Supplementary Fig. 16). This procedure was repeated for each aberration type to construct the complete wavefront profile.

### Aberration correction of 3D WFM images

To correct aberrations in 3D WFM images, we applied magnitude modulation to the pupil function, specifically for SA. When the refractive index of the sample is lower than that of the immersion medium, two angle-dependent effects—reflection losses and wavefront compression—alter the effective magnitude^37^. At the sample–coverslip interface, the electromagnetic field amplitude decreases due to Fresnel reflection but increases near the periphery due to wavefront compression of the emitted light. Total magnitude change is modeled as the product of these two effects.

We incorporated this into the pupil function as:

15 $${P}^{{\prime} {\prime} }\left(u,v,z,\psi ,\varepsilon \left(\psi \right)\right)=P\left(u,v\right){B}^{{\prime} }\left(u,v,\Delta {n}_{2}\right){{{\rm{e}}}}^{{{\rm{i}}}\frac{2{{\rm{\pi }}}}{\lambda }\mathop{\sum }\limits_{\psi =1}^{n\psi }\left\{{W}_{a}\left[u,v,\psi ,\varepsilon \left(\psi \right)\right]{W}_{d}\left(u,v,z\right){W}_{t}\left(u,v,\psi ,\varepsilon \left(\psi \right)\right)\right\}}$$

where:

- ${B}^{{\prime} }$ is the total magnitude change,
- $\Delta {n}_{2}$ is the refractive index mismatch between the sample and the ideal condition,
- $\varepsilon (\psi )$ is the optimized aberration magnitude for type $\psi$.

Because ∆*n*_*2*_ is not directly measured, we estimated it by comparing wavefronts generated with known ∆*n*_*1*_ values (immersion medium mismatch) and selecting the ∆*n*_*2*_ that best matched the wavefront at a fixed depth (5 µm).

Using the modified pupil function, we computed aberrated PSF:

16 $${h}^{{\prime} {\prime} }\left(x,y,z,\psi ,\varepsilon \left(\psi \right)\right)={{{\mathscr{F}}}}_{2D}^{-1}\left[{P}^{{\prime} {\prime} }\right]\cdot {{{\mathscr{F}}}}_{2D}^{-1}{\left[{P}^{{\prime} {\prime} }\right]}^{* }$$

The corresponding 3D OTF was obtained via:

17 $${O}^{{\prime} {\prime} }\left({k}_{x},{k}_{y},{k}_{z},\psi ,\varepsilon \left(\psi \right)\right)={{{\mathscr{F}}}}_{3D}\left[{h}^{{\prime} {\prime} }\right]$$

From this, the MTF and PTF were extracted:

18 $${{O}^{{\prime} {\prime} }=A}^{{\prime} {\prime} }\left({k}_{x},{k}_{y},{k}_{z},\psi ,\varepsilon \left(\psi \right)\right){{{\rm{e}}}}^{{{\rm{i}}}{{{ \varnothing }}}^{{\prime} {\prime} }\left({k}_{x},{k}_{y},{k}_{z},\psi ,\varepsilon \left(\psi \right)\right)}$$

The final deconvolution was performed using both amplitude and phase components:

19 $${D}^{{\prime} {\prime} }\left({k}_{x},{k}_{y},{k}_{z},\psi ,\varepsilon \left(\psi \right)\right)=\left\{\begin{array}{cc}\frac{A\left({k}_{x},{k}_{y},{k}_{z}\right)D\left({k}_{x},{k}_{y},{k}_{z}\right)}{{A}^{{\prime} {\prime} }\left({k}_{x},{k}_{y},{k}_{z},\psi ,\varepsilon \left(\psi \right)\right){{{\rm{e}}}}^{{{\rm{i}}}{{{ \O }}}^{{\prime} {\prime} }\left({k}_{x},{k}_{y},{k}_{z},\psi ,\varepsilon \left(\psi \right)\right)}+{w}^{2}}, & \left({k}_{x} < {k}_{{xmax}},{k}_{y} < {k}_{{ymax}},{k}_{z} < {k}_{{zmax}}\right)\\ 0, \hfill & \left({k}_{x}\ge {k}_{{xmax}},{k}_{y}\ge {k}_{{ymax}},{k}_{z}\ge {k}_{{zmax}}\right)\end{array}\right.$$

where $A$ is the ideal MTF, and *w* = 0.01 is the Wiener filter constant.

The corrected image was obtained via inverse 3D Fourier transform:

20 $${d}^{{\prime} {\prime} }\left(x,y,z,\psi ,\varepsilon \left(\psi \right)\right)={{{\mathscr{F}}}}_{3D}^{-1}\left[{D}^{{\prime} {\prime} }\right]$$

### Measurement of optical aberrations in 3D-SIM

We assumed a conventional three-beam configuration for 3D-SIM^2^. Aberration measurement was performed using two complementary approaches.

#### Pseudo-WFM-based measurement

Raw 3D-SIM images were summed across all illumination phases and rotations to generate Pseudo-WFM images. These high-SNR images were then processed using the same phase-based deconvolution method described for WFM (see **“Wavefront measurement from 3D WFM images”**).

#### Modulation amplitude optimization

To directly measure aberrations from raw 3D-SIM data, we implemented an optimization procedure based on modulation amplitude. The 3D-SIM reconstruction algorithm was adapted from the original source code (“XYenhance3d”) written by Gustafsson et al.^2^, rewritten in Python, and modified to accommodate both theoretical and measured OTFs.

During reconstruction, we fitted the modulation depth ${c}_{m}$ and initial phase ${\varphi }_{m}$ for each illumination angle and interference order $m$ (where $m={\mathrm{0,1,2}}$). The modulation amplitude ${c}_{m}{{{\rm{e}}}}^{{{{\rm{i}}}\varphi }_{m}}$ was calculated as the peak of the cross-correlation between frequency components ${D}_{0}\left(k\right)\cdot {O}_{m}^{{\prime} }\left(k+{\mu }_{m}\right)$ and ${D}_{m}\left(k+{\mu }_{m}\right)\cdot {O}_{0}^{{\prime} }\left(k\right)$, where:

- ${D}_{0}$ and ${D}_{m}$ are the frequency components of the 0^th^- and $m$^th^-order image data,
- ${O}_{0}^{{\prime} }$ and ${O}_{m}^{{\prime} }$ are the aberrated OTFs for the respected order,
- ${\mu }_{m}$ is the lateral shift vector for the $m$^th^-order.

The modulation depth was then computed as ${\left|{c}_{m}{{{\rm{e}}}}^{{{{\rm{i}}}\varphi }_{m}}\right|}^{2}$.

To isolate the effect of aberrations, we varied only the phase components ${ \varnothing }^{{\prime} }$ of the OTF:

21 $${O}_{m}^{{\prime} }\left(k,\psi ,\alpha \right)=A\left(k\right){{{\rm{e}}}}^{{{\rm{i}}}{{{ \varnothing }}}^{{\prime} }\left(k,\psi ,\alpha \right)}$$

where $A(k)$ is the ideal MTF and ${ \varnothing }^{{\prime} }$ encodes the aberration. This approach mirrors the phase-only deconvolution used in WFM.

For aberrations exhibiting horizontal asymmetry, we selected datasets corresponding to illumination angles perpendicular to the aberration axis to improve measurement accuracy.

### Creation of the theoretical 3D-SIM OTF

To generate a theoretical 3D-SIM PSF incorporating optical aberrations, we first constructed an illumination pupil function ${P}_{{ill}}\left(u,v,r,s\right)$ composed of three Gaussian spots. Each spot had a standard deviation of 0.4 pixels and was placed at positions determined by the spatial frequency vector ${\mu }_{m}$:

22 $$l\left(u,v,n,r,b\right)={nb}\frac{{\mu }_{m}\left({k}_{x},{k}_{y}\right)}{m}$$

where:

- $n=-{\mathrm{1,0}},+1$ denotes the diffraction order,
- $r$ is the illumination angle,
- $b$ is the zoom coefficient of the pupil.

The phase of $n=\pm 1$ spots was adjusted to $\pm \frac{2{{\rm{\pi }}}s}{5}$ relative to the central spot ($n=0$), where $s={\mathrm{0,1,2,3,4}}$ represents the phase index.

To incorporate aberrations, the illumination pupil was modified as:

23 $${P}_{{ill}}^{{\prime} }\left(u,v,z,\psi ,\alpha ,r,s\right)={P}_{{ill}}\left(u,v,r,s\right){{{\rm{e}}}}^{{{\rm{i}}}\frac{2{{\rm{\pi }}}}{\lambda }\left\{\mathop{\sum }\limits_{\psi =1}^{n\psi }\left[{W}_{a}\left(u,v,\psi ,\alpha \right)+{W}_{d}\left(u,v,z\left(\psi ,\alpha \right)\right)+{W}_{t}\left(u,v,x\left(\psi ,\alpha \right),y\left(\psi ,\alpha \right)\right)\right]\right\}}$$

The corresponding 2D illumination intensity profile was computed as:

24 $${i}^{{\prime} }\left(x,y,z,\psi ,\alpha ,r,s\right)={{{\mathscr{F}}}}_{2D}^{-1}\left[{P}_{{ill}}^{{\prime} }\right]\cdot {{{{\mathscr{F}}}}_{2D}^{-1}\left[{P}_{{ill}}^{{\prime} }\right]}^{* }$$

A 3D illumination profile was generated by stacking 2D slices across defocus positions. This illumination profile was then multiplied by the aberrated WFM PSF to obtain the 3D-SIM PSF:

25 $${h}_{{SIM}}^{{\prime} }\left(x,y,z,\psi ,\alpha ,r,s\right)={i}^{{\prime} }\left(x,y,z,\psi ,\alpha ,r,s\right)\cdot {h}^{{\prime} }\left(x,y,z,\psi ,\alpha \right)$$

The OTFs for each interference orders were obtained by applying a 3D Fourier transform to the corresponding PSFs, with frequency components separated as previously described^1,2,29^.

In practice, measured OTFs yielded better reconstruction quality than theoretical ones. Therefore, we adjusted the measured OTFs to match the target image dimensions using third-order spline interpolation, padding, or cropping. If the measured OTF was radially averaged, it was polar-transformed to recover full dimensionality. For aberration measurement, the phase component (PTF) of the measured OTF was replaced with a theoretical PTF.

### Subregional operations

To perform subregional aberration correction in 3D images, we manually selected regions of interest for wavefront measurement. Each subregion was processed using a pupil size larger than 256 × 256 pixels to avoid aliasing artifacts in the frequency domain.

For correction, the full image was divided into segments corresponding to the selected subregions. Each voxel was assigned to the nearest subregion based on Euclidean distance from the subregion boundaries. Because target regions often have irregular shapes unsuitable for direct processing, we enclosed each in the smallest possible cubic volume and apply aberration correction within that cube.

After correction, binary masks were used to isolate the corrected regions, and all corrected target regions were merged to reconstruct the full image.

In 3D-SIM, the procedure was similar but required additional care. Small target regions often failed to yield accurate estimates of the structured illumination wave vector (line spacing, angle, amplitude, and phase). Therefore, while aberrations were measured in small subregions, correction were applied to the entire image using the wave vector estimated from a larger region. Region masks were used to restrict the correction spatially. Although this approach increased processing time, it significantly improved the accuracy of SIM reconstruction parameters.

### Simulation for the accuracy of aberration measurement

To evaluate the accuracy of aberration measurement (Fig. 2a–d, Supplementary Figs. 8), we simulated a single PSF using the following parameters:

- Objective lens NA: 1.42
- Emission wavelength: 528 nm
- Image size: 128 × 128 × 128 pixels (X × Y × Z)
- Pixel size: 267 × 92 × 92 nm
- Pupil size: 512 × 512 pixels

The simulated PSFs included either Seidel or Zernike aberrations, and the peak intensity was normalized to 100.

To simulate noise, we added Poisson-distributed noise with a mean value $\mu$ defined as:

26 $${{\rm{\mu }}}={\left(\frac{100}{{SNR}}\right)}^{2}$$

The final noisy image was generated as

27 $$J\left(x,y,z,\psi ,\alpha ,{SNR}\right)=100\frac{{h}^{{\prime} }\left(x,y,z,\psi ,\alpha \right)}{\max \left[{h}^{{\prime} \left(x,y,z,\psi ,\alpha \right)}\right]}+\rho \left[{{\rm{\mu }}}\left({SNR}\right)\right]$$

where:

- $h{\prime}$ is the aberrated PSF,
- $\rho \left(\mu \right)$ is a Poisson noise function with mean µ.

Images of the fiber-like structures (Fig. 2e) were generated using a second-order Bézier curve defined by three control points in three-dimensional space (x, y, z), denoted as ${C}_{i}\left(i={\mathrm{0,1,2}}\right)$. The curve function $B\left(p\right)$, parametrized by $p\in \left[{\mathrm{0,1}}\right]$, is expressed as:

28 $$s\left(p\right)={C}_{0}+p\left({C}_{1}-{C}_{0}\right)$$

29 $$t\left(p\right)={C}_{1}+p\left({C}_{2}-{C}_{1}\right)$$

30 $$B\left(p\right)=s\left(p\right)+p\left(t\left(p\right)-s\left(p\right)\right)={\left(1-p\right)}^{2}{C}_{0}+2\left(1-p\right)p{C}_{1}+{p}^{2}{C}_{2}$$

where $s\left(p\right)$ and $t\left(p\right)$ lie on the line segments connecting ${C}_{0}-{C}_{1}$ and ${C}_{1}-{C}_{2}$, respectively. The point $B\left(p\right)$ is then defined on the line segment between $s\left(p\right)$ and $t\left(p\right)$. For random generation of fibrous structures, the positions of the control points ${C}_{i}$ were sampled from a uniform distribution within the specified spatial domain. Fifty fibers with a radius of 180 nm were generated for the simulation, assuming a pixel size of 90 × 90 × 270 nm (X × Y × Z). To reduce sampling along the Z-axis, two out of every three optical sections were removed, resulting in a threefold reduction in Z-axis sampling. The simulated fiber images were convolved with theoretical PSFs corresponding to an objective lens with NA 1.42 and an emission wavelength of 528 nm, with or without optical aberrations.

The ∅CAO algorithm was then applied to these images to estimate the aberration parameters. The results were compared to the ground truth to assess measurement accuracy.

### Sample preparation

#### Fluorescent beads

Yellow-green-fluorescent beads (FluoSpheres, 0.1 µm, F8803, Thermo Fisher Scientific) were diluted 1:1000 in ethanol. Immediately after dilution, 5 µL of the suspension was spread onto clean 18 × 18 mm coverslips (No. 1H, Matsunami Glass Ind.) and allowed to dry. The coverslips were mounted using Prolong^TM^ Gold Antifade Mountant (Thermo Fisher Scientific) and placed on glass slides.

#### Bead slide with plant tissue

A glass slide with grid arrays (R1L3S3P, Thorlabs) was overlaid with a 50 µm-thick silicone sheet (Mitsubishi Plastics), from which a small piece of the sheet was excised with a razor blade to make a 50 µm-deep chamber. 0.1% polyethyleneimine (PEI) was spread on the center of the slide chamber and was incubated at room temperature for 3 min. After washing with distilled water and drying, yellow-green-fluorescent beads (FluoSpheres, 0.1 µm, F8803, Thermo Fisher Scientific) were diluted 1: 5000 in distilled water, were spread on the center of the slide chamber, and were incubated at room temperature for 3 min. After washing with distilled water and drying, the chamber was filled with BCDAT liquid medium^41^. A leaf segment was excised from a 4-week-old gametophore of *P. patens* ssp. *patens* (the Gransden strain) cultured on BCDAT agar medium. The leaf was placed in the chamber filled with BCDAT liquid medium, and a coverslip (No. 1, Matsunami Glass Ind.) was put on the chamber.

#### HeLa cells

HeLa cells (Riken Cell Bank, RCB007) were cultured on glass-bottom culture dishes (MatTek Life Sciences) and fixed with 3.7% formaldehyde (POLY SCIENCE, INC.) for 15 min at room temperature. After three washes with phosphate-buffered saline (PBS), the cells were permeabilized with 0.1% Triton X−100 in PBS for 5 min and washed three times with PBS.

For actin staining, Alexa Fluor^TM^ 488 phalloidin (Thermo Fisher Scientific) was diluted to 1:100 in 1% bovine serum albumin (BSA) in PBS and incubated with the cells for 1 h at room temperature. After three PBS washes, cells were mounted in Prolong^TM^ Gold.

For tubulin immunostaining, cells were blocked with 1% BSA in PBS for 1 h. Primary antibodies against α-tubulin (TAT−1^42^) was diluted 1:50 in 1% BSA in PBS and incubated overnight at 4 °C. After four PBS washes, Alexa Fluor^TM^ 488-conjugated secondary antibody (Thermo Fisher Scientific) was applied at 1:500 dilution for 3 h at room temperature. Cells were washed three times with PBS and mounted in Prolong^TM^ Gold.

#### *C. elegans* (WFM imaging)

Strain XA3501(unc−119(ed3) ruls32 III; ojls1) was maintained using standard methods^43^. Adult worms were anesthetized with 0.7% sodium azide in a glass-bottomed dish (MatTek Life Sciences) for imaging.

For Supplementary Fig. 14, *C. elegans* nematodes of genotype *mGFP::dsb−1* (from ref. ^44^ but with an additional mutation, A206K, to promote monomericity) were synchronized at the L4 larval stage and then grown for a further 24 h at 20 ˚C in the dark. For imaging, 5−10 worms were picked into 15 μl of M9 buffer (42.3 mM Na_2_HPO_4_, 22.0 mM KH_2_PO_4_, 85.6 mM NaCl, 1.0 mM MgSO_4_; pH 7.0, containing 0.1% tricaine and 0.01% levamisole) on a 22 × 22 mm #1S micro cover glass (Matsunami). The sample was then placed on a glass slide (Matsunami Inc, cat #S9901) and quickly sealed with VaLaP (1:1:1 mixture of vaseline, lanolin and paraffin) previously melted at 65 ˚C.

#### *C. elegans* (3D-SIM immunofluorescence)

Adult wild-type N2 worms were dissected in 15 µL of EBT buffer (HEPES pH7.4, 27.5 mM, NaCl 129.8 mM, KCl 52.8 mM, EDTA 2.2 mM, EGTA 0.55 mM, 0.1% Tween-20, 0.15% tricaine, 0.015% levamisole). Dissected germlines were fixed by addition of 15 µL of fixative solution (EBT with 2% formaldehyde lacking Tween) for 2 min. After freeze-cracking and incubation in 100% methanol at −25 °C for 1 min, slides were washed for 10 min in PBS with 1% Tween-20 (PBST).

Slides were blocked in PBST supplemented with 0.5% BSA for 30 min, followed by overnight incubation with two primary antibodes^45^, guinea pig anti-SYP−1 and rabbit anti-SYP-1(T452p) diluted 1:100 in PBST, at 4 °C in a humid chamber. Slides were then washed in PBST for 10 min, followed by incubation with secondary antibodies (anti-guinea pig Dylight488 and anti-rabbit Dylight594, Jackson ImmunoResearch, both at 1:500 dilution) at room temperature in a humid chamber for 2 h. The slides were then washed for 10 min in PBST and incubated a further 10 min in PBST with 1 µg mL^−1^ of 4’,6-diamidino-2-phenylindole (DAPI) to stain DNA. After a final 10 min in PBS, 13 µL of freshly prepared mounting medium (50 µL 5 M TRIS base in 450 µL of 4% *n*-propyl gallate glycerol) was added to the sample under a #1-S micro coverslip (Matsunami Glass Ind.) and the sample was sealed with nail polish.

#### Image acquisition

WFM and 3D-SIM were performed using a DeltaVision OMX SR microscope (Leica Microsystems) equipped with multiple pco.edge 5.5 sCMOS cameras (Excelitas Technologies Corp.). Imaging was conducted using either an oil immersion objective lens (PLAPON 60XO NA1.42; Olympus) or a silicone immersion objective lens (UPLSAPO 60XS NA1.30; Olympus). Detection channels covered the blue (419–450 nm), green (504–552 nm), and orange (590–627 nm) emission ranges, with excitation laser lines of 405, 488, and 561 nm. The temperature around the sample stage was 27 ± 1 °C. The immersion oils used in this study had the following refractive indices (at 23 °C) and the Abbe numbers:

- Olympus: 1.406 (*v*_e_ = 52),
- Cargille: 1.500 (*v*_D_ = 35.3), 1.504 (*v*_D_ = 35.0), 1.506 (*v*_D_ = 34.8), 1.508 (*v*_D_ = 34.7), 1.510 (*v*_D_ = 34.5), 1.512 (*v*_D_ = 34.4), 1.514 (*v*_D_ = 34.2), 1.516 (*v*_D_ = 34.1), and 1.518 (*v*_D_ = 33.9).

### Image processing software

Multichannel image alignment and 3D drift correction were performed using *Chromagnon* (v0.95)^46^, with the target image as the reference. Deconvolution was applied to original or ∅CAO-corrected images using a theoretical unaberrated PSF. Scaled Heavy Ball Richardson-Lucy deconvolution was implemented with *Deconwolf* (v0.4.5)^47^ for 30 iterations for Fig. 4c and 50 iterations for Supplementary Fig. 13c using the “--gpu” option on Ubuntu 22.04LTS with an NVIDIA RTX A6000 GPU. The enhanced Gold method^48^ was performed by the IVE *Priism* suite (v4.2.7)^49^ for 12 iterations with a Wiener filter enhancement factor 0.8 using the “enhanced ratio” option on CentOS 6. Conventional Richardson-Lucy deconvolution was carried out with *DeconvolutionLab2* (v2.1.2)^50^ for 60 iterations on Ubuntu 22.04LTS.

### Reporting summary

Further information on research design is available in the Nature Portfolio Reporting Summary linked to this article.

## Supplementary information

Supplementary information

Description of Additional Supplementary Files

Supplementary movie 1

Reporting Summary

## Data Availability

The image data generated in this study are available in Zenodo with the identifier 10.5281/zenodo.15826325.

## Code availability

Demonstration executable software of ∅CAO is available in Zenodo with the identifier 10.5281/zenodo.15042907.

## References

[1] Gustafsson, M. G. L. Surpassing the lateral resolution limit by a factor of two using structured illumination microscopy. *J. Microsc.* **198**, 82–87 (2000). 10810003 10.1046/j.1365-2818.2000.00710.x

[2] Gustafsson, M. G. L. et al. Three-dimensional resolution doubling in wide-field fluorescence microscopy by structured illumination. *Biophys. J* **94**, 4957–4970 (2008). 18326650 10.1529/biophysj.107.120345 PMC2397368

[3] Schermelleh, L. et al. Subdiffraction multicolor imaging of the nuclear periphery with 3D structured illumination microscopy. *Science* **320**, 1332–1336 (2008). 18535242 10.1126/science.1156947 PMC2916659

[4] Sheppard, C. J. R. Super-resolution in confocal imaging. *Optik* **80**, 53–54 (1988).

[5] Müller, C. B. & Enderlein, J. Image scanning microscopy. *Phys. Rev. Lett.* **104**, 198101 (2010). 20867000 10.1103/PhysRevLett.104.198101

[6] York, A. G. et al. Resolution doubling in live, multicellular organisms via multifocal structured illumination microscopy. *Nat. Methods* **9**, 749–754 (2012). 22581372 10.1038/nmeth.2025 PMC3462167

[7] Hell, S. W. & Wichmann, J. Breaking the diffraction resolution limit by stimulated emission: stimulated-emission-depletion fluorescence microscopy. *Opt. Lett.* **19**, 780–782 (1994). 19844443 10.1364/ol.19.000780

[8] Betzig, E. et al. Imaging intracellular fluorescent proteins at nanometer resolution. *Science* **313**, 1642–1645 (2006). 16902090 10.1126/science.1127344

[9] Rust, M. J., Bates, M. & Zhuang, X. Sub-diffraction-limit imaging by stochastic optical reconstruction microscopy (STORM). *Nat. Methods* **3**, 793–795 (2006). 16896339 10.1038/nmeth929 PMC2700296

[10] Chen, F., Tillberg, P. W. & Boyden, E. S. Expansion microscopy. *Science* **347**, 543–548 (2015). 25592419 10.1126/science.1260088 PMC4312537

[11] Demmerle, J. et al. Strategic and practical guidelines for successful structured illumination microscopy. *Nat. Protoc.* **12**, 988–1010 (2017). 28406496 10.1038/nprot.2017.019

[12] Hampson, K. M. et al. Adaptive optics for high-resolution imaging. *Nat. Rev. Methods Primers* **1**, 1–26 (2021). 10.1038/s43586-021-00066-7 PMC8892592 35252878

[13] Tao, X., Azucena, O. & Kubby, J. Adaptive optical microscopy using guide star–based direct wavefront sensing. in *Wavefront Shaping for Biomedical Imaging* (eds Kubby, J., Gigan, S. & Cui, M.) 22–57. 10.1017/9781316403938.003. (Cambridge University Press, 2019)

[14] Booth, M. J. Adaptive optical microscopy: the ongoing quest for a perfect image. *Light Sci. Appl.* **3**, e165–e165 (2014).

[15] Wu, J. et al. Iterative tomography with digital adaptive optics permits hour-long intravital observation of 3D subcellular dynamics at millisecond scale. *Cell* **184**, 3318–3332.e17 (2021). 34038702 10.1016/j.cell.2021.04.029

[16] Antonello, J., Andrade, D. & Booth, M. Adaptive optical microscopy using image-based wavefront sensing. in *Wavefront Shaping for Biomedical Imaging* (eds Kubby, J., Gigan, S. & Cui, M.) 3–21. 10.1017/9781316403938.002. (Cambridge University Press, 2019).

[17] Wang, J. et al. Deep3DSIM: super-resolution imaging of thick tissue using 3D structured illumination with adaptive optics. *eLife* **14**, e102144 (2025). 41150055 10.7554/eLife.102144 PMC12563541

[18] Wang, J. & Zhang, Y. Adaptive optics in super-resolution microscopy. *Biophys. Rep* **7**, 267–279 (2021). 37287764 10.52601/bpr.2021.210015 PMC10233472

[19] Stockham, T. G., Cannon, T. M. & Ingebretsen, R. B. Blind deconvolution through digital signal processing. *Proc. IEEE* **63**, 678–692 (1975).

[20] Ayers, G. R. & Dainty, J. C. Iterative blind deconvolution method and its applications. *Opt. Lett.* **13**, 547–549 (1988). 19745959 10.1364/ol.13.000547

[21] Markham, J. & Conchello, J.-A. Parametric blind deconvolution: a robust method for the simultaneous estimation of image and blur. *J. Opt. Soc. Am. A* **16**, 2377–2391 (1999). 10.1364/josaa.16.002377 10517022

[22] Holmes, T. J., Biggs, D. & Abu-Tarif, A. Blind deconvolution. in *Handbook Of Biological Confocal Microscopy* (ed. Pawley, J. B.) 468–487. 10.1007/978-0-387-45524-2_24. (Springer US, 2006)

[23] Kam, Z., Hanser, B., Gustafsson, M. G. L., Agard, D. A. & Sedat, J. W. Computational adaptive optics for live three-dimensional biological imaging. *Proc. Natl. Acad. Sci. USA* **98**, 3790–3795 (2001). 11274396 10.1073/pnas.071275698 PMC31131

[24] Edrei, E. & Scarcelli, G. Memory-effect based deconvolution microscopy for super-resolution imaging through scattering media. *Sci. Rep.* **6**, 33558 (2016). 27633483 10.1038/srep33558 PMC5025711

[25] Zhang, P. et al. Deep learning-driven adaptive optics for single-molecule localization microscopy. *Nat. Methods* **20**, 1748–1758 (2023). 37770712 10.1038/s41592-023-02029-0 PMC10630144

[26] Liu, S. et al. Universal inverse modeling of point spread functions for SMLM localization and microscope characterization. *Nat. Methods* **21**, 1082–1093 (2024). 38831208 10.1038/s41592-024-02282-x PMC12330227

[27] Guo, M. et al. Deep learning-based aberration compensation improves contrast and resolution in fluorescence microscopy. *Nat. Commun* **16**, 313 (2025). 39747824 10.1038/s41467-024-55267-x PMC11697233

[28] Tamada, Y. et al. Optical property analyses of plant cells for adaptive optics microscopy. *Int. J. Optomechatron.* **8**, 89–99 (2014).

[29] Chen, X. et al. Superresolution structured illumination microscopy reconstruction algorithms: a review. *Light Sci Appl* **12**, 172 (2023). 37433801 10.1038/s41377-023-01204-4 PMC10336069

[30] Zhuang, Y. & Shi, X. Expansion microscopy: a chemical approach for super-resolution microscopy. *Curr. Opin. Struct. Biol.* **81**, 102614 (2023). 37253290 10.1016/j.sbi.2023.102614 PMC11103276

[31] Hümpfer, N., Thielhorn, R. & Ewers, H. Expanding boundaries – a cell biologist’s guide to expansion microscopy. *J. Cell. Sci.* **137**, jcs260765 (2024). 38629499 10.1242/jcs.260765 PMC11058692

[32] Wu, J. et al. An integrated imaging sensor for aberration-corrected 3D photography. *Nature* **612**, 62–71 (2022). 36261533 10.1038/s41586-022-05306-8 PMC9712118

[33] Kang, I., Zhang, Q., Yu, S. X. & Ji, N. Coordinate-based neural representations for computational adaptive optics in widefield microscopy. *Nat. Mach. Intell.* **6**, 714–725 (2024).

[34] Alshaabi, T. et al. Fourier-based three-dimensional multistage transformer for aberration correction in multicellular specimens. *Nat. Methods* **22**, 2171–2179 (2025). 41034611 10.1038/s41592-025-02844-7 PMC12510882

[35] Johnson, C. et al. Phase-diversity-based wavefront sensing for fluorescence microscopy. *Optica* **11**, 806–820 (2024).

[36] Gerchberg, R. W. & Saxton, W. O. A practical algorithm for the determination of phase from image and diffraction plane pictures. *Optik* **35**, 237–246 (1972).

[37] Hanser, B. M., Gustafsson, M. G. L., Agard, D. A. & Sedat, J. W. Phase-retrieved pupil functions in wide-field fluorescence microscopy. *J. Microsc.* **216**, 32–48 (2004). 15369481 10.1111/j.0022-2720.2004.01393.x

[38] Gibson, S. F. & Lanni, F. Experimental test of an analytical model of aberration in an oil-immersion objective lens used in three-dimensional light microscopy. *J. Opt. Soc. Am. A* **9**, 154–166 (1992). 1738047 10.1364/josaa.9.000154

[39] Li, J., Xue, F. & Blu, T. Fast and accurate three-dimensional point spread function computation for fluorescence microscopy. *J. Opt. Soc. Am. A* **34**, 1029–1034 (2017). 10.1364/JOSAA.34.001029 29036087

[40] Sheppard, C. J. R., Gu, M., Kawata, Y. & Kawata, S. Three-dimensional transfer functions for high-aperture systems. *J. Opt. Soc. Am. A* **11**, 593–598 (1994).

[41] Nishiyama, T., Hiwatashi, Y., Sakakibara, K., Kato, M. & Hasebe, M. Tagged mutagenesis and gene-trap in the moss, Physcomitrella patens by shuttle mutagenesis. *DNA Res.* **7**, 9–17 (2000). 10718194 10.1093/dnares/7.1.9

[42] Woods, A. et al. Definition of individual components within the cytoskeleton of Trypanosoma brucei by a library of monoclonal antibodies. *J. Cell Sci.* **93**, 491–500 (1989). 2606940 10.1242/jcs.93.3.491

[43] Brenner, S. The genetics of Caenorhabditis elegans. *Genetics* **77**, 71–94 (1974). 4366476 10.1093/genetics/77.1.71 PMC1213120

[44] Guo, H. et al. Phosphoregulation of DSB-1 mediates control of meiotic double-strand break activity. *eLife* **11**, e77956 (2022). 35758641 10.7554/eLife.77956 PMC9278955

[45] Sato-Carlton, A., Nakamura-Tabuchi, C., Chartrand, S. K., Uchino, T. & Carlton, P. M. Phosphorylation of the synaptonemal complex protein SYP-1 promotes meiotic chromosome segregation. *J. Cell Biol.* **217**, 555–570 (2017). 29222184 10.1083/jcb.201707161 PMC5800814

[46] Matsuda, A., Schermelleh, L., Hirano, Y., Haraguchi, T. & Hiraoka, Y. Accurate and fiducial-marker-free correction for three-dimensional chromatic shift in biological fluorescence microscopy. *Sci. Rep.* **8**, 7583 (2018). 29765093 10.1038/s41598-018-25922-7 PMC5954143

[47] Wernersson, E. et al. Deconwolf enables high-performance deconvolution of widefield fluorescence microscopy images. *Nat. Methods* **21**, 1245–1256 (2024). 38844629 10.1038/s41592-024-02294-7 PMC11239506

[48] Agard, D. A., Hiraoka, Y., Shaw, P. & Sedat, J. W. Fluorescence microscopy in three dimensions. *Methods Cell Biol.* **30**, 353–377 (1989). 2494418 10.1016/s0091-679x(08)60986-3

[49] Chen, H., Hughes, D. D., Chan, T.-A., Sedat, J. W. & Agard, D. A. IVE (Image Visualization Environment): a software platform for all three-dimensional microscopy applications. *J. Struct. Biol.* **116**, 56–60 (1996). 8742723 10.1006/jsbi.1996.0010

[50] Sage, D. et al. DeconvolutionLab2: an open-source software for deconvolution microscopy. *Methods* **115**, 28–41 (2017). 28057586 10.1016/j.ymeth.2016.12.015

