# Supplementary material for: Phase-based computational adaptive optics enables artifact-free super-resolution microscopy
Source: Commun Eng. 2026 Mar 9;5:75. doi: 10.1038/s44172-026-00622-7 (PMC13100136; doi:10.1038/s44172-026-00622-7)
Supplement: Supplementary file 1 — Supplementary information [file 44172_2026_622_MOESM1_ESM.pdf]

Supplementary information

Phase-Based Computational Adaptive Optics Enables Artifact-Free Super-Resolution Microscopy

Atsushi Matsuda[1*], Carlos Mario Rodriguez-Reza[2], Yosuke Tamada[3,4,5], Yamato Matsuo[1], Takaharu G. Yamamoto[1], Takako Koujin[1], Peter M. Carlton[2,6]

[1] Advanced ICT Research Institute, National Institute of Information and Communications Technology, Kobe 651-2492, Japan

[2] Graduate School of Biostudies, Kyoto University, Kyoto 606-8501, Japan

[3] School of Engineering, Utsunomiya University, Utsunomiya, 321-8585 Japan

[4] National Institute for Basic Biology, Okazaki, 444-8585 Japan

[5] Basic Biology Program, SOKENDAI, Okazaki, 444-8585 Japan

[6] Radiation Biology Center, Kyoto University, Yoshida-Konoecho, Kyoto 606-8501, Japan

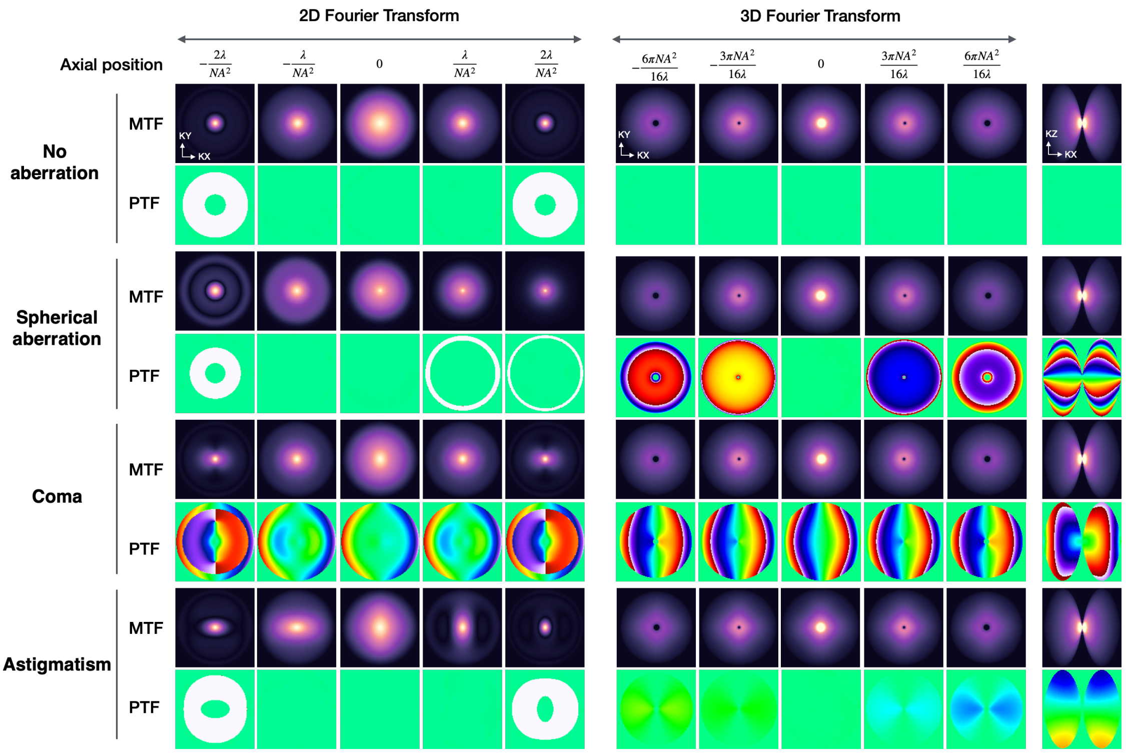

Supplementary Fig. 1. 2D or 3D optical transfer functions (OTFs) of wide-field microscopy (WFM) with and without optical aberrations.

2D OTFs were generated by applying 2D Fourier transformation of 2D point spread functions (PSFs) at the focal planes indicated at the top. 3D OTFs were obtained via 3D Fourier transformation of 3D PSFs and are shown at selected axial frequencies in the top and side views, centered at zero frequency along the Y-axis. The introduced aberration magnitudes were 0.003 for spherical aberration (Δn_1), 0.29 for coma, and 0.26 for astigmatism. Modulation transfer functions (MTFs) are displayed using a gamma value of 0.4 to enhance visibility of low-intensity features. “PTF” denotes “phase transfer function”.

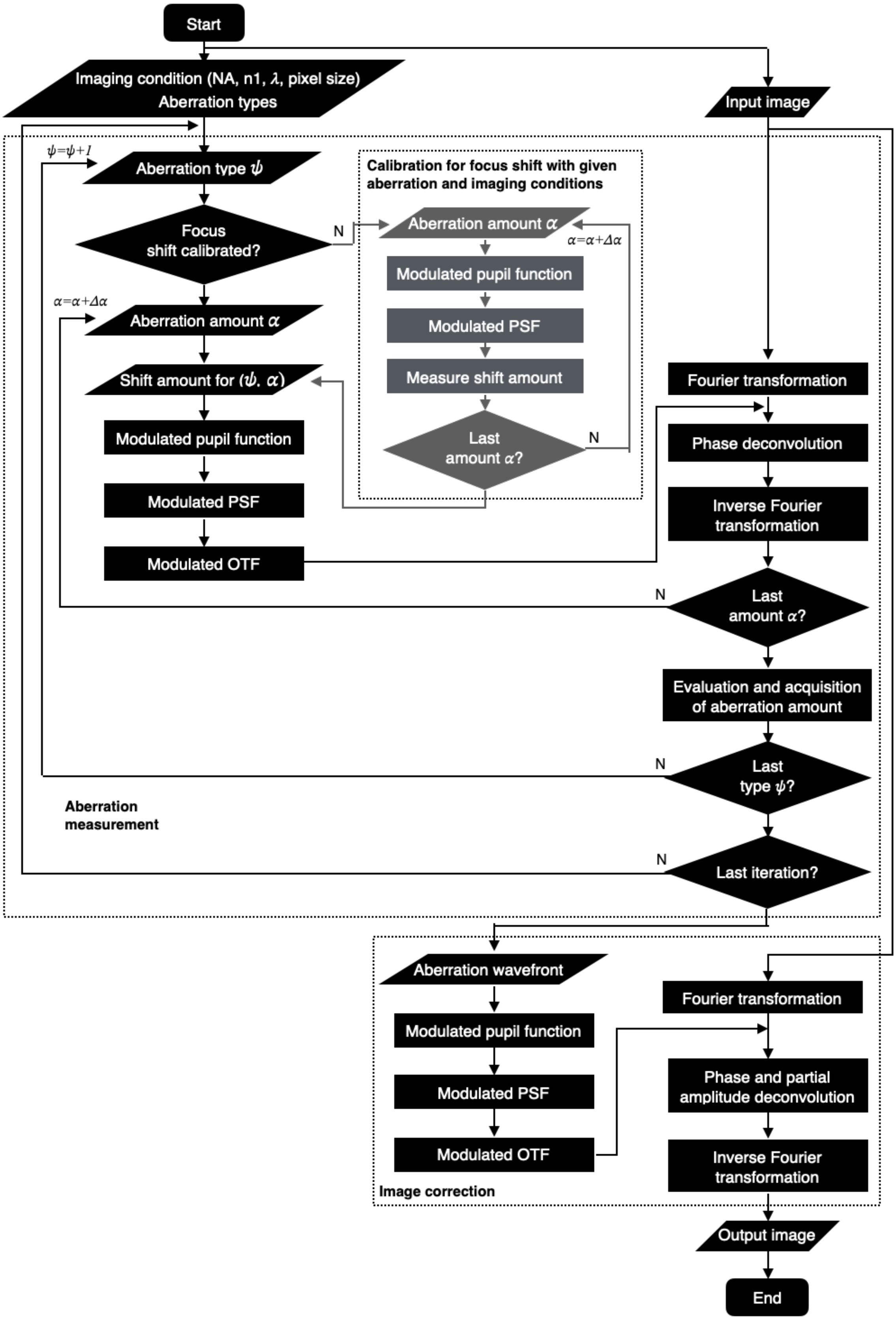

Supplementary Fig. 2. Flowchart of øCAO.

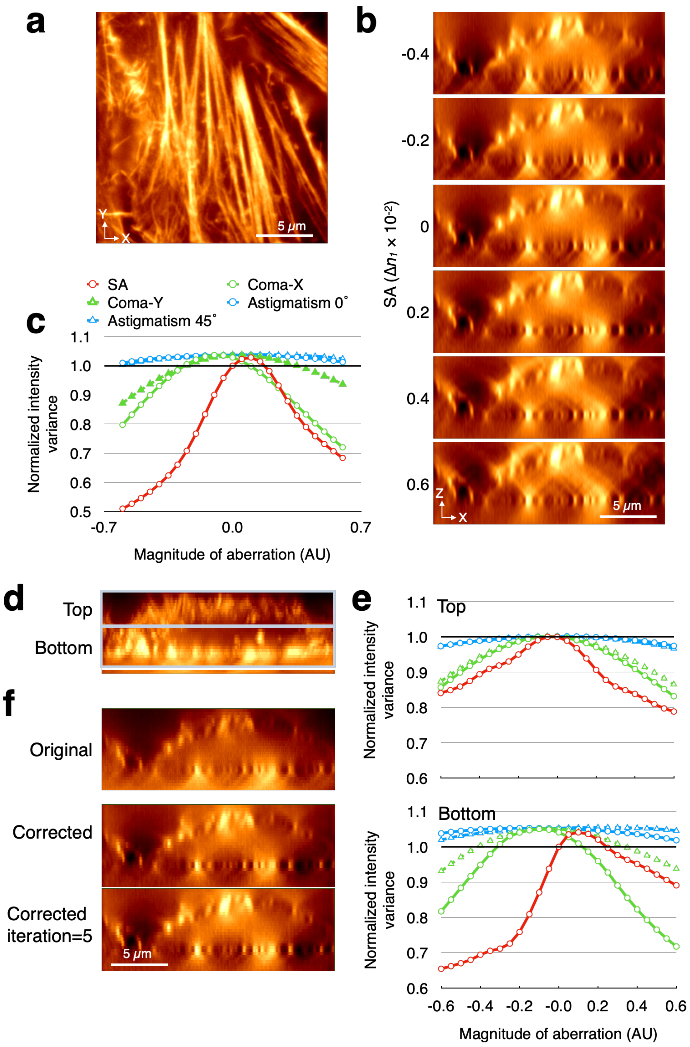

Supplementary Fig. 3. Measurement of spherical aberrations (SA) in actin filaments images in HeLa cells.

a Top view of the original image from a single optical section. **b** Side views corresponding to **a**, shown as single section along the Y-axis with varying degrees of SA introduced via phase transfer function (PTF) deconvolution. **c** Image quality scores plotted against introduced aberration levels. **d.** The image stack was divided into “Top” and “Bottom” regions, and Seidel aberrations were measured independently in each. **e.** Image quality scores as a function of aberration level for “Top” and “Bottom” regions, which exhibited different magnitudes. **f.** Side views of original and corrected images for “Top” and “Bottom” regions. Corrected images display more symmetric intensity pattern than the originals. Images from five iterations are also shown, appearing similar to those corrected without iteration.

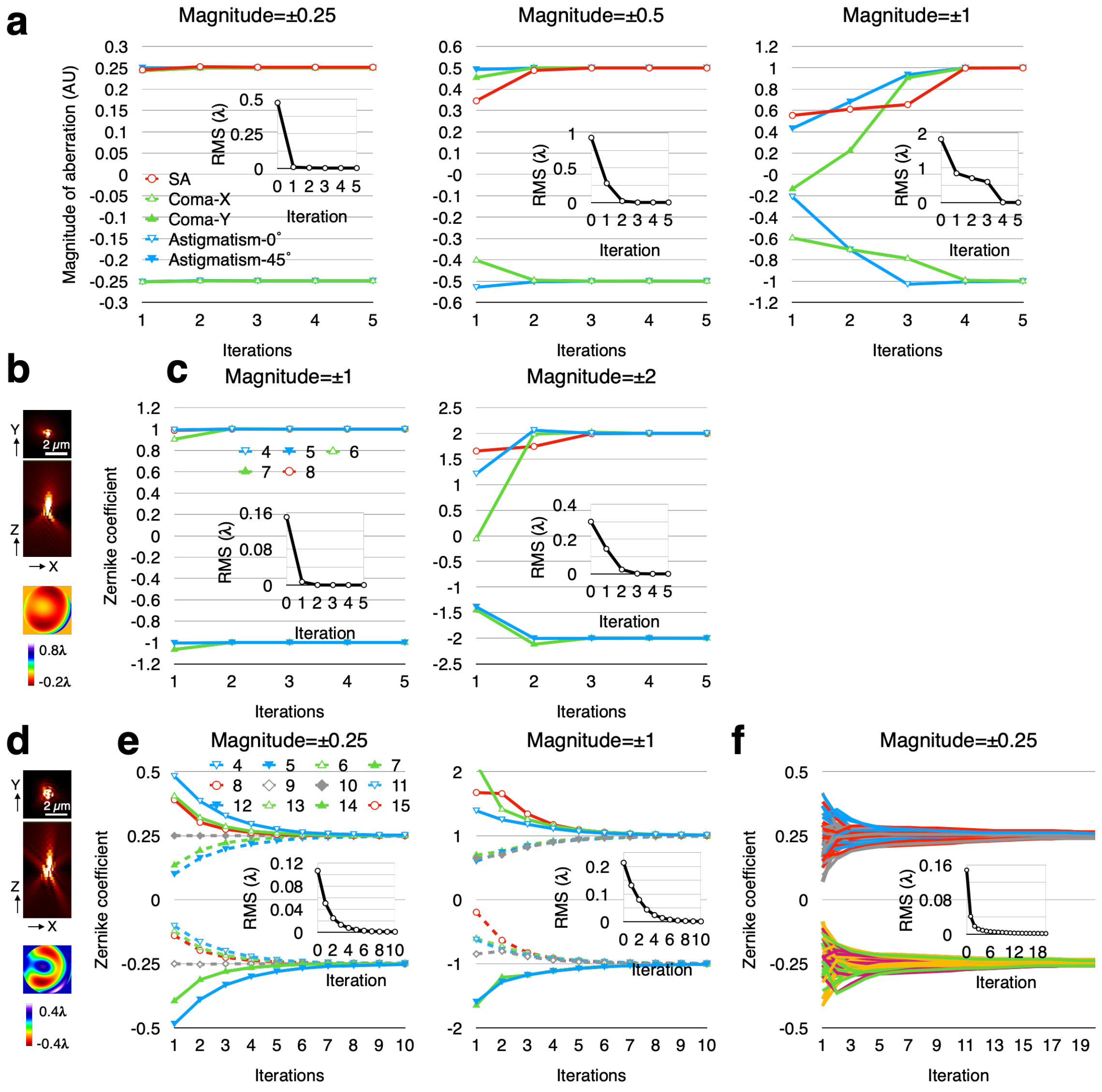

Supplementary Fig. 4. Iterative estimation of optical aberrations using øCAO.

a, c, e, f Plots of aberration magnitude estimated by øCAO across iterations. Ground-truth values are indicated above each plot, alternating between positive and negative as in Fig. 2b and 2d. Insets show wavefront root mean square (RMS) error in units of wavelength. Zero iteration corresponds to no correction. **a** Sidel aberrations; **c** first-order Zernike aberrations; **e** first and second-order Zernike aberrations; **f** first- to eighth-order Zernike aberrations (legend omitted for clarity). **b, d** 3D point objects with Zernike aberrations and corresponding wavefronts. Images of point objects are displayed with a gamma value of 0.5 to enhance low-intensity features. **b** First-order Zernike aberrations of magnitude ±1 (even modes positive, odd modes negative). **d**

first- to eighth-order Zernike aberrations of magnitude ±0.25 (even modes positive, odd modes negative).

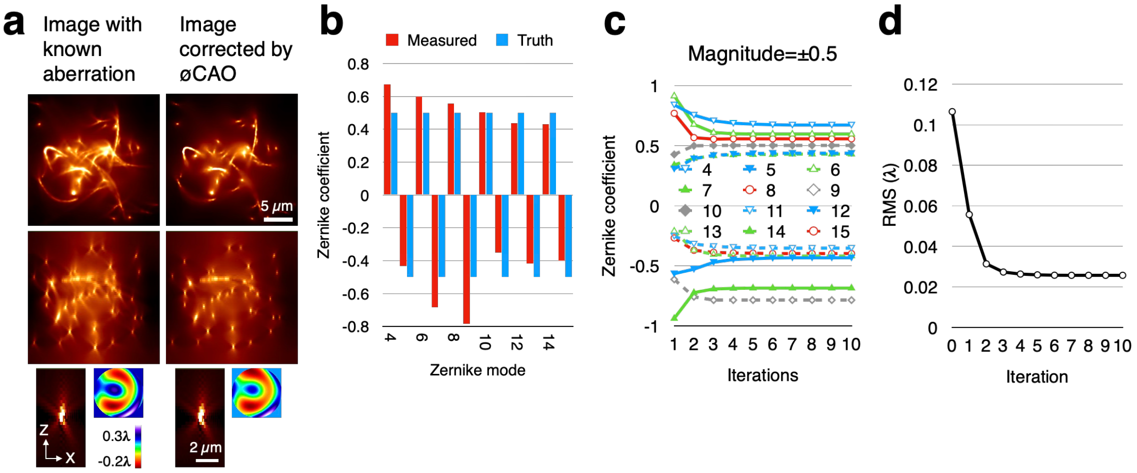

Supplementary Fig. 5. Estimation of complex Zernike modes in simulated fibrous structures using øCAO.

a Top and side views of simulated fibrous structures with known aberrations and after øCAO correction. True wavefront and estimated wavefronts and point spread functions (PSFs) are shown below. **b**. Measured versus ground-truth Zernike coefficients. **c**. Zernike coefficients estimated by øCAO across iterations. Ground-truth values are ±0.5 (even modes positive, odd modes negative). **d**. Wavefront root mean square (RMS) error in units of wavelength. Zero iteration corresponds to no correction.

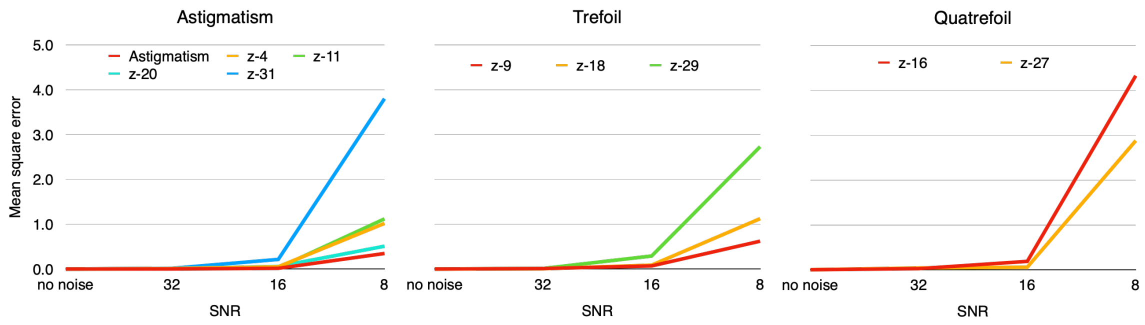

Supplementary Fig. 6. Effect of signal-to-noise ratio (SNR) on the accuracy of Seidel and Zernike mode measurements.

Simulation results showing the measurement accuracy of astigmatism, trefoil, quatrefoil and their corresponding Zernike modes (denoted as “z-“ followed by Wyant indices) under varying SNR conditions. Normalized mean square error of the measured aberration values is plotted as a function of SNR.

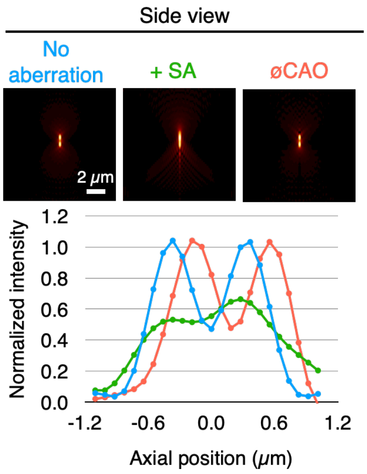

Supplementary Fig. 7. øCAO resolves closely spaced point sources.

Simulated theoretical point spread functions (PSFs) were positioned 0.7 μm apart along the optical axis. After introducing spherical aberration (SA) (Δn_1=0.7×10^{-2}), the two points appeared fused. However, following correction with øCAO using Seidel aberrations, two points were successfully resolved. Axial intensity profiles corresponding to the images are shown below.

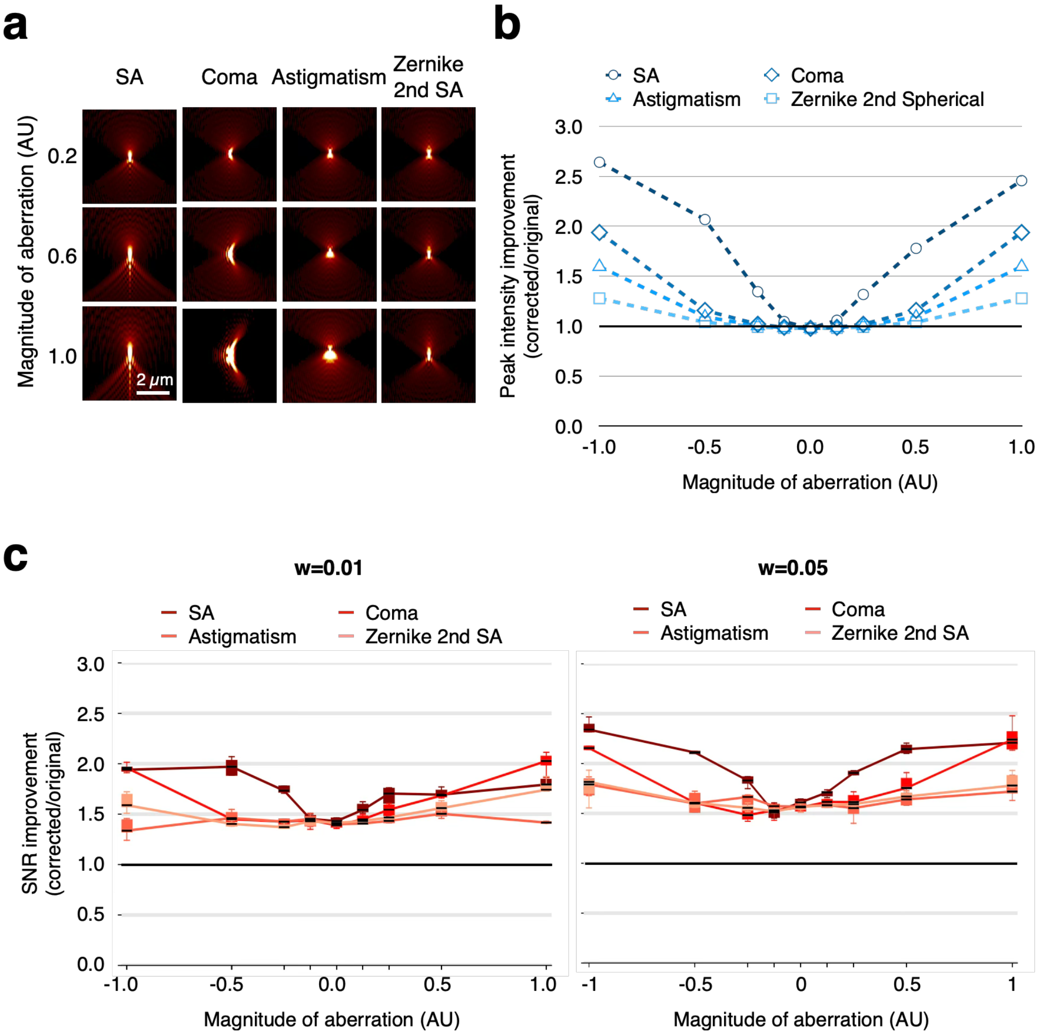

Supplementary Fig. 8. Simulation-based evaluation of signal-to-noise ratio (SNR) improvement following optical aberration correction.

a Side views of point spread functions (PSFs) with various types and magnitudes of optical aberrations. Each PSF images consists of 128 optical sections sampled at the Nyquist frequency. “SA” denotes spherical aberration. Images are displayed with a gamma value of 0.4 to enhance low-intensity features. **b** Improvement in the peak intensity in noise-free images following aberration correction. **c** Box plots of SNR improvement in noisy images in five independent simulation experiments.

In the presence of noise, SNR improvement by øCAO is attributed to partial modulation transfer function (MTF) deconvolution and denoising via bandpass filtering. The effectiveness of deconvolution also depends on the Wiener filter constant, which was set to 0.01 (w=0.01) in this study. Results obtained with a higher constant (w=0.05) are

also shown. For further details, see Supplementary Note 1.

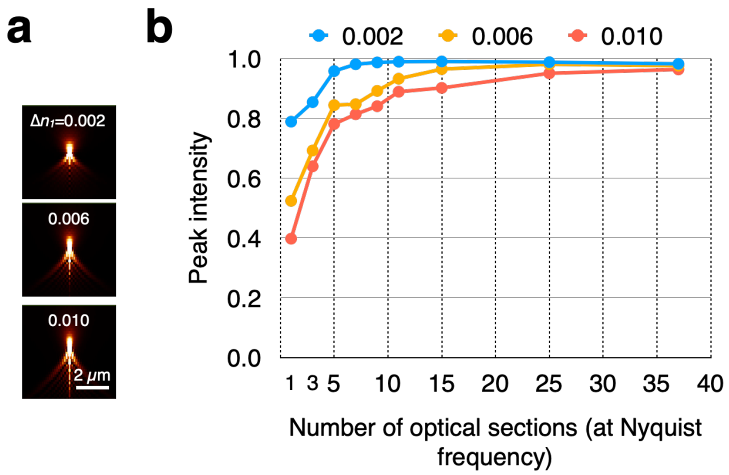

Supplementary Fig. 9. Simulation of peak intensity recovery as a function of the number of optical sections.

a Side views of simulated point spread functions (PSFs) with varying degrees of spherical aberration (Δn_1). **b** Normalized peak intensity after aberration correction, plotted as a function of the number of optical sections at the Nyquist sampling frequency of the imaging system. The three curves correspond to different SA levels shown in **a**. Data points at section "1" represent the original, uncorrected intensity.

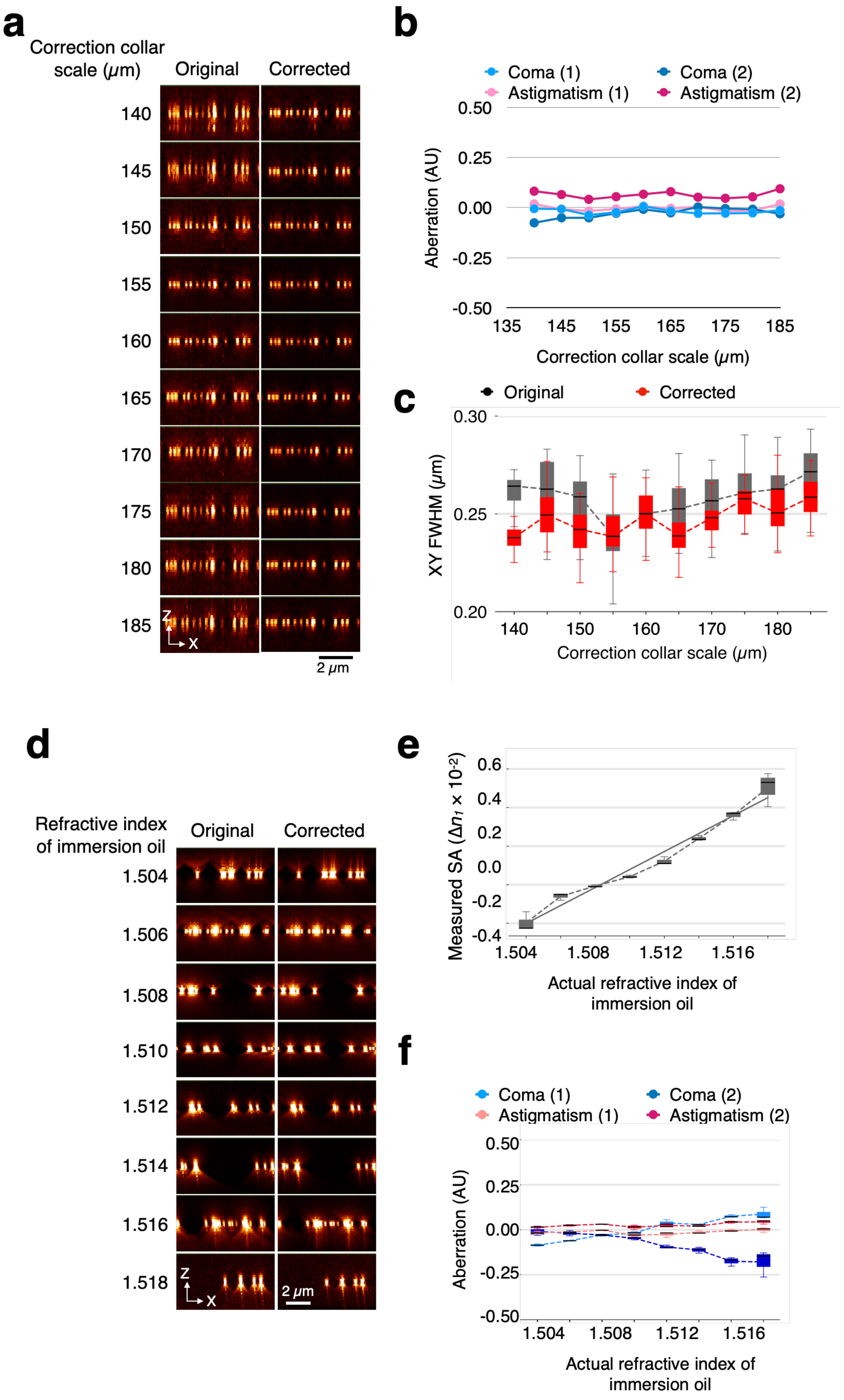

Supplementary Fig. 10. Accurate measurement of introduced spherical aberration (SA) in yellow-green 100-nm fluorescent bead images using øCAO.

a Side views of 3D stacks from the same field of view, sequentially acquired using a silicone immersion objective lens with varying correction collar positions. Images are shown before and after aberration correction using øCAO with Seidel aberrations. **b** Measured coma and astigmatism plotted as functions of the correction collar scale. Coma (1) and (2) represent aberrations along the X- and Y-axes, respectively; astigmatism (1) and (2) represent aberrations along the X- and its orthogonal direction. All non-SA aberrations remained minimal. **c** Box plots of horizontal full width at half maximums (FWHM) of 100-nm beads ($n \geq 10$), before and after aberration correction. **d** Side views of 3D stacks from different fields of view, sequentially acquired using an oil-immersion objective lens with varying refractive indices of immersion oil. Images are shown before and after correction using øCAO with Seidel aberrations. **e** A box plot of measured SA as a function of the refractive index of immersion oil from three independent images. **f** Box plots of measured coma and astigmatism as functions of the refractive index of immersion oil from three independent images.

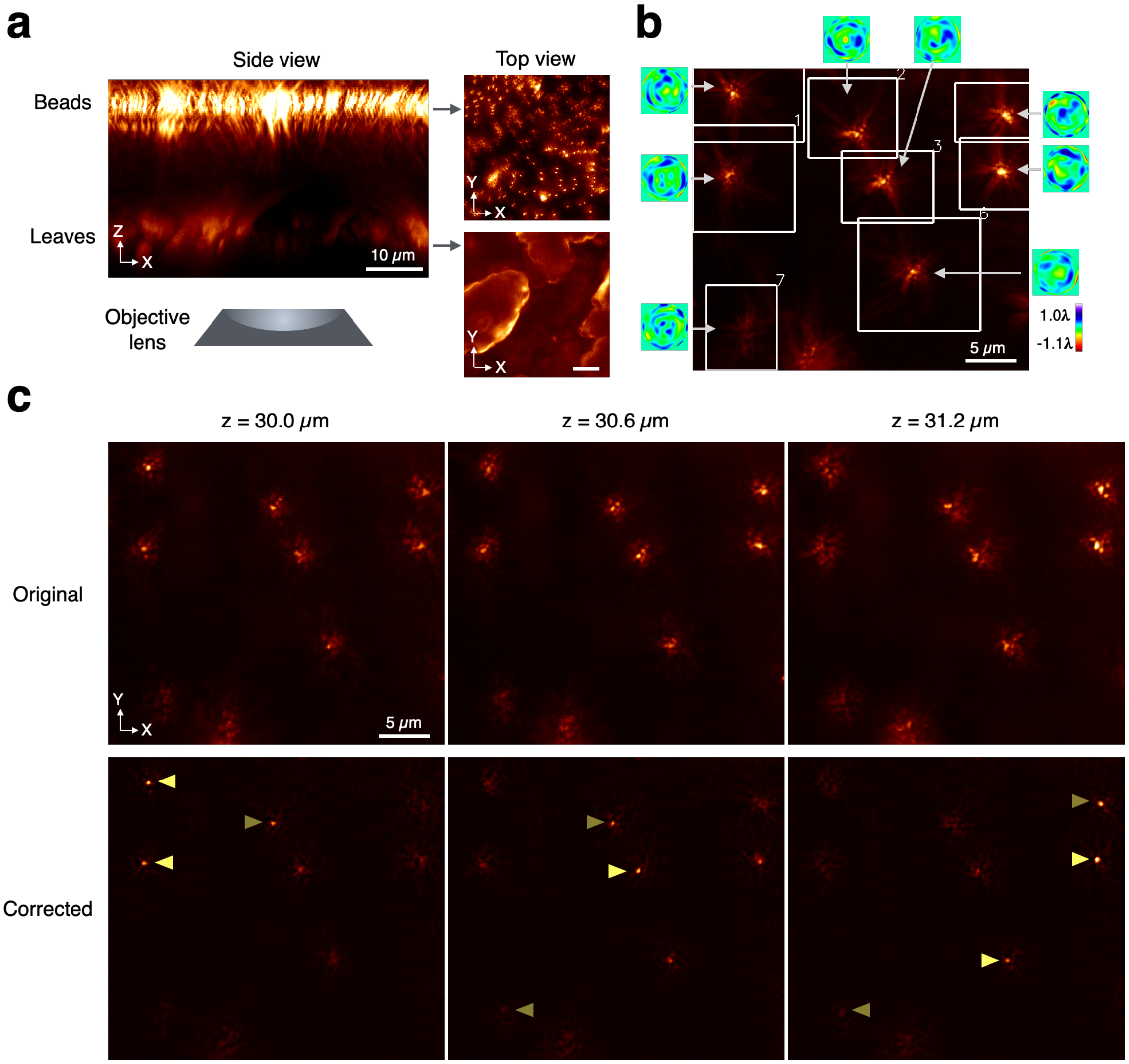

Supplementary Fig. 11. øCAO corrects highly aberrated bead images.

a Side-view maximum intensity projection of the entire 3D image of the sample slide. The sample comprises plant tissue at the bottom (shown as autofluorescence) and fluorescent beads on the glass surface at the top (approximately z = 30 μm from the bottom). **b** Maximum intensity projection of the original beads image. Subregions used for aberration measurement are marked with white boxes, each accompanied by the corresponding measured wavefront. Wavefronts were estimated using 96 Zernike modes (Wyant indices 4-99). Notably, even adjacent subregions exhibit markedly different wavefronts. **c** Representative optical sections of the original and corrected bead images. Yellow arrows indicate beads in focus, while gray arrows denote beads located between focal planes.

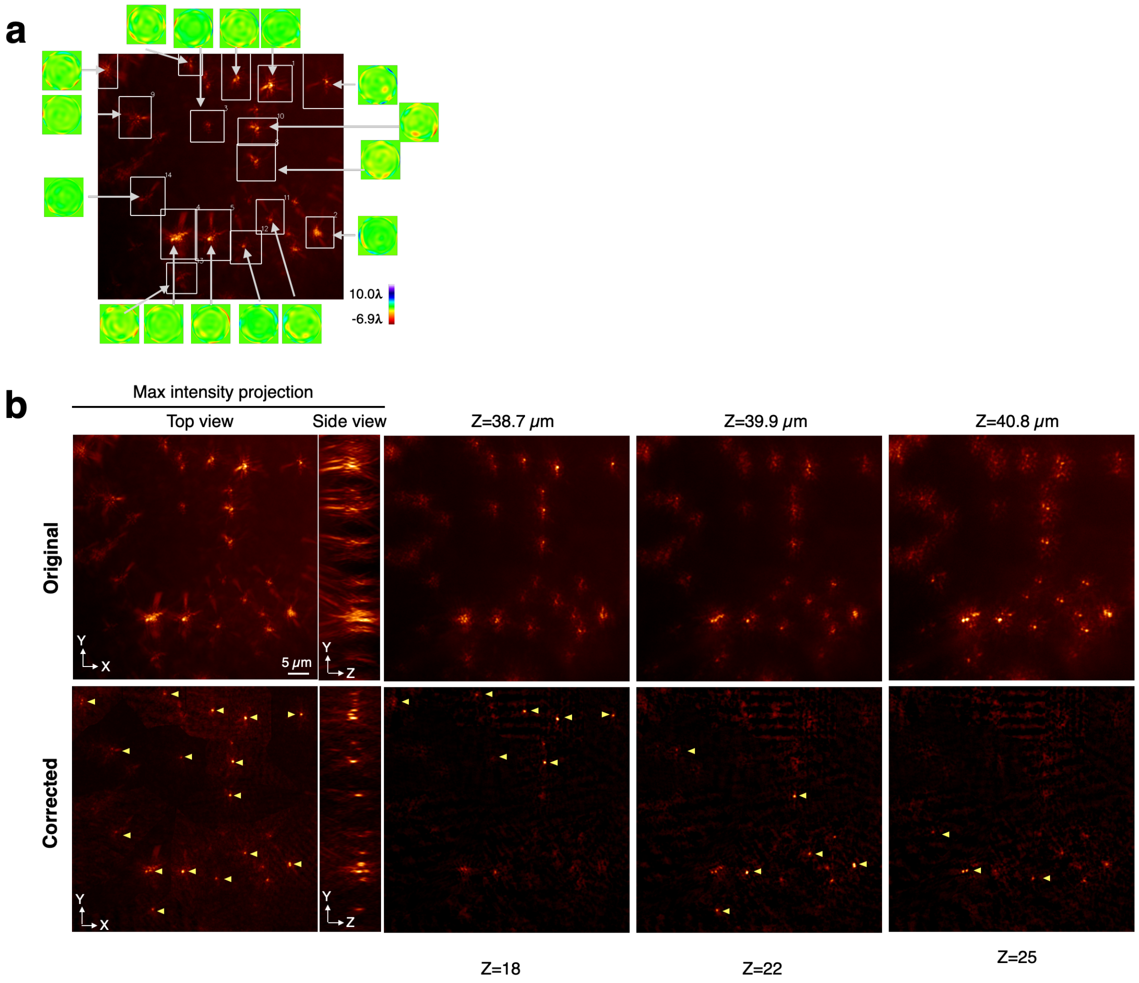

Supplementary Fig. 12. øCAO substantially corrects extremely aberrated bead images.

a Maximum intensity projection of the original bead image acquired from the same sample as Supplementary Fig. 11, but from a different field of view. Subregions used for aberration measurement are marked with white boxes, each accompanied by the corresponding measured wavefront. Wavefronts were estimated using 96 Zernike modes (Wyant indices 4-99) over 10 iterative correction cycles. **b** Maximum intensity projection and representative optical sections of the original and corrected bead images. Yellow arrows indicate beads used for wavefront estimation.

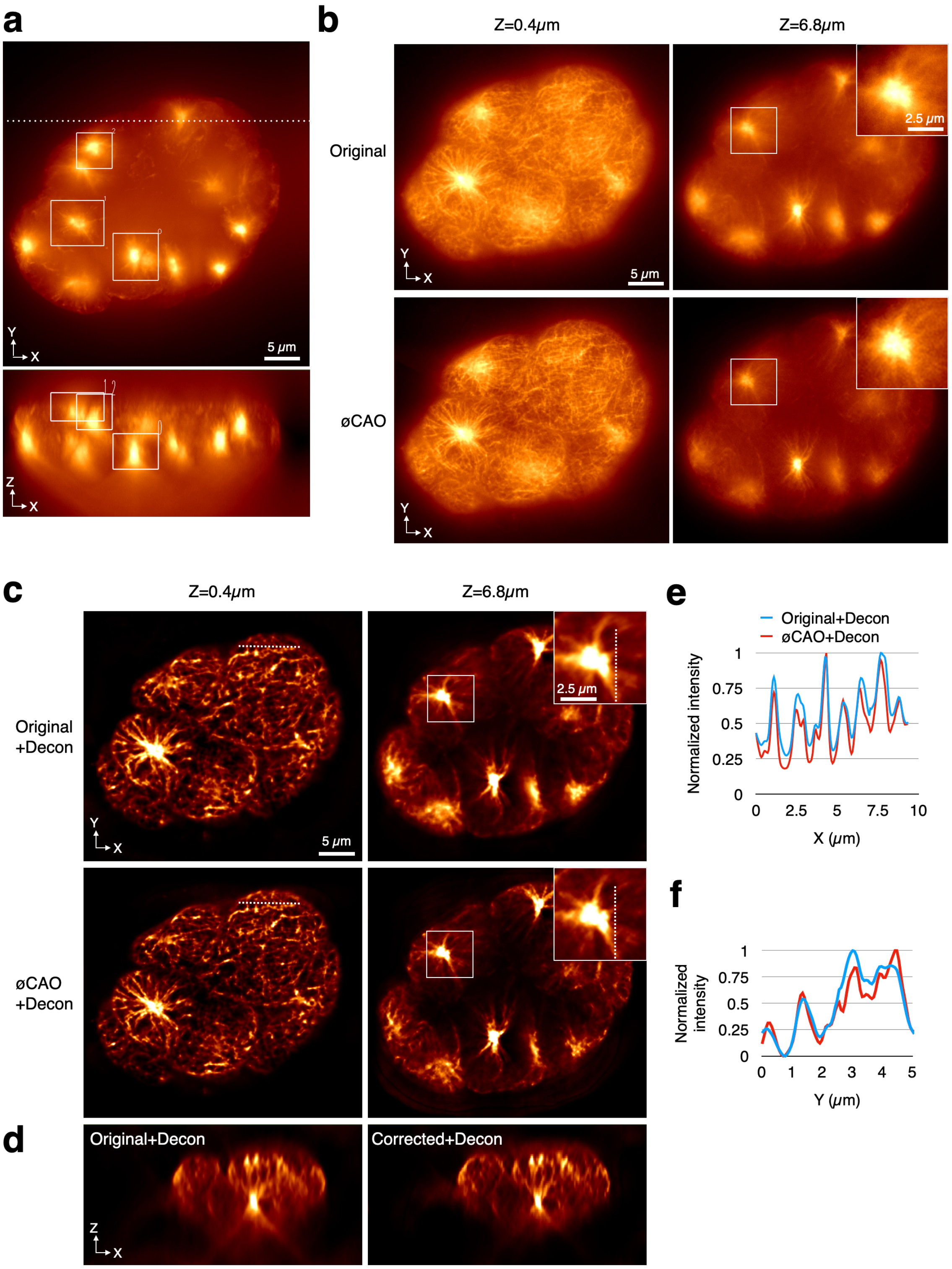

Supplementary Fig. 13. øCAO improves deconvolution efficiency in 3D reference datasets.

a Maximum-intensity projection of a publicly available *C. elegans* embryo image (FITC channel) (source: https://bigwww.epfl.ch/deconvolution/bio/). Subregions used for aberration measurement are marked with white boxes. **b, c** Representative single optical sections of the original and corrected images: (**b**) after Seidel aberration correction and (**c**) after deconvolution using the Richardson-Lucy algorithm with Scaled Heavy Ball optimization. Axial positions at the top indicate approximate distance from the bottom of the animal. Boxed regions are shown magnified two-fold in the insets. **d** Side views of the deconvolved images at a single Y-axis section indicated by the dotted line in **a**. **e**, **f** Line profiles of the original and øCAO-corrected deconvolved images. Profiles in **e** correspond to dotted lines in left panels of **c**, and profiles in **f** correspond to dotted lines in the inset of the right panels in **c**.

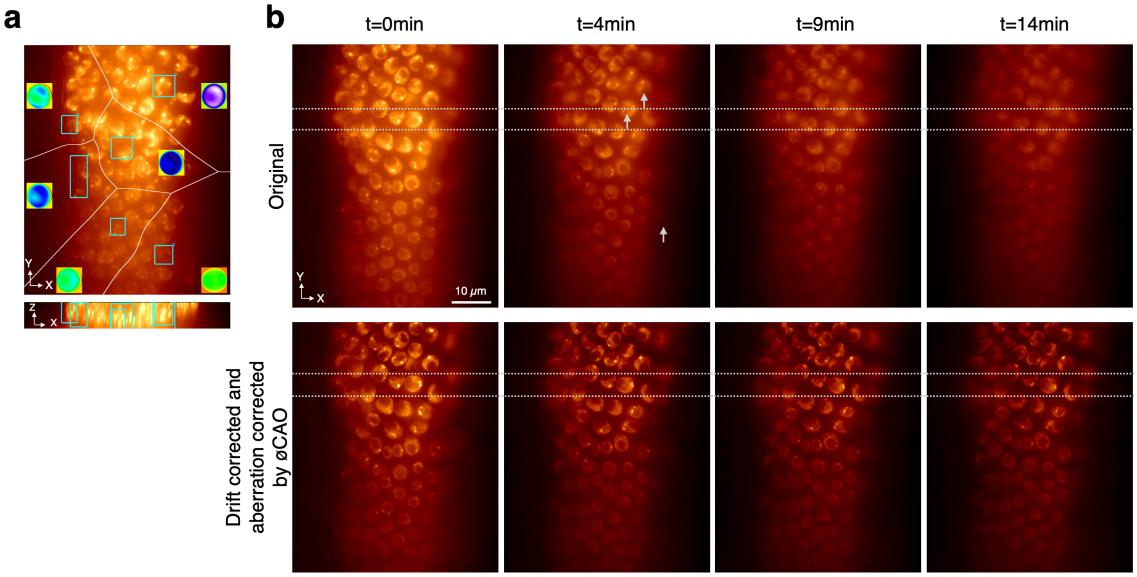

Supplementary Fig. 14. Regional øCAO correction applied after sample drift compensation.

a Meiotic chromosomes labeled with mGFP-DSB-1 were imaged in a live *C. elegans* adult hermaphrodite. Z stacks were acquired every minute using immersion oil with a refractive index of 1.500 (suboptimal compared to the ideal 1.513) to introduce artificial spherical aberration. Subregions for aberration measurement were selected at time 0 min and are indicated by blue boxes. Measured wavefronts from each region are shown adjacent to the boxes and were applied to surrounding areas outlined in white. **b** Representative optical sections at selected time points from the original and drift-compensated, aberration-corrected images. The worm exhibited movement along the Y axis between 0 and 4 minutes (note the shift in nuclei positions relative to the dotted lines), and along the Z axis at later time points (9 and 14 minutes) resulting in defocusing. After computational drift correction, øCAO was applied, constantly restoring resolution and signal-to-noise ratio (SNR) across the entire image.

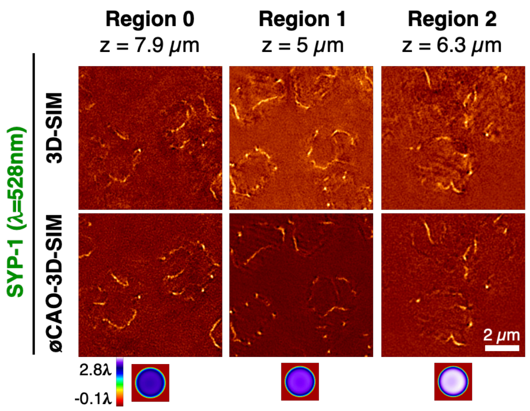

Supplementary Fig. 15. øCAO restores optimal resolution in 3D-SIM.

Reconstructed 3D-SIM images in optimized green (528 nm) channel. Representative images from three subregions (indicated in Fig. 4**h**) are shown. Wavefronts used for correction are displayed under the corresponding panels.

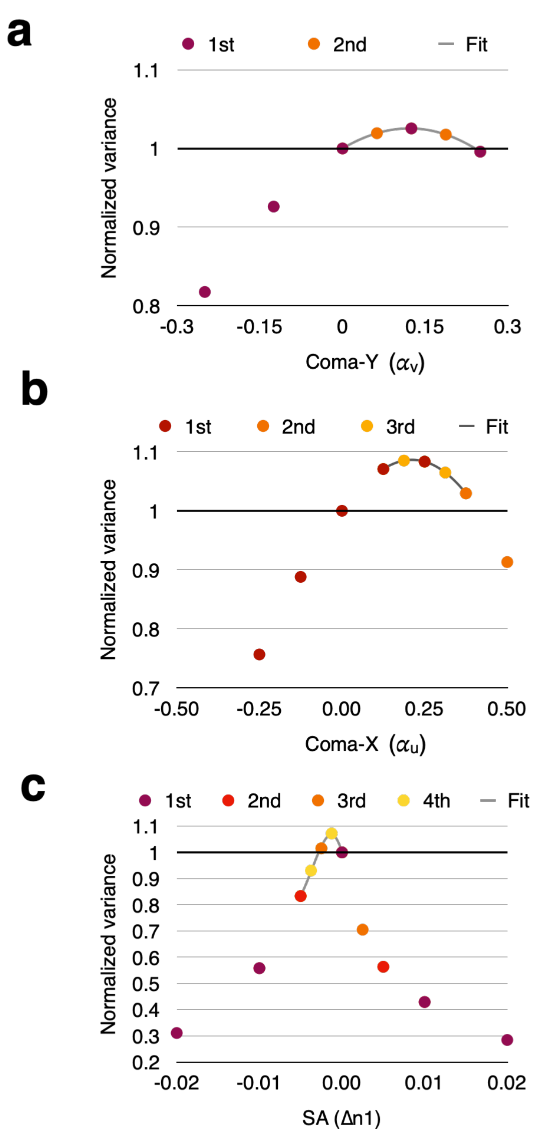

Supplementary Fig. 16. Illustration of convex optimization in øCAO.

Normalized variance plots after phase deconvolution of fluorescent bead samples (see Fig. 1**d**, **e**). **a** Example where the optimal value lies within the initial five sampled aberration magnitudes (dark dots labeled “1st”; the aberration type: coma-Y). In the second step, two additional sampling points were added near the magnitude yielding the highest variance (orange dots labeled “2nd”). Finally, a polynomial curve was fitted using five sampling points near the peak (curved line labeled “Fit”). **b** Example where the optimal value was outside of the initial sampling range. When the highest-variance magnitude occurred at the edge of the initial sampling points, two additional points were added (orange dots labeled “2nd”) and the process was repeated until the optimal

value was within the sampling range. The same procedure as in **a** was then applied. **c**. For spherical aberration (SA), even when the peak variance was within the initial sampling range, three additional sampling steps were required, because the SA peak was too sharp for accurate prediction using a simple polynomial fit.

Supplementary Note 1

In the final step of øCAO, modulation transfer function (MTF) alterations caused by optical aberrations were corrected alongside phase transfer function (PTF) adjustment to achieve full aberration compensation (see Methods and Supplementary Fig. 2). In this study, MTF correction was restricted to aberration-induced changes to isolate the effect of computational adaptive optics.

While PTF deconvolution redistributes image intensity without altering brightness, MTF deconvolution amplifies both signal and noise. The greater the aberration, the more pronounced the MTF distortion, and, thus the stronger its influence during correction. Consequently, in images with substantial noise and aberration, achieving high signal-to-noise ratio (SNR) may be challenging.

In noise-free conditions, peak intensity improvement was solely dependent on the magnitude of aberration (Supplementary Fig. 8b), and computational adaptive optics performed equivalently to optical methods. However, under noisy conditions, øCAO partially amplifies both signal and noise via MTF deconvolution, modulated by the Wiener filter constant (set to 0.01 in this study). Additionally, øCAO applies bandpass filtering to remove frequencies beyond the theoretical limit, yielding an approximate 1.5-fold SNR enhancement.

The combined effect of these presences resulted in an overall SNR improvement of 1.5-2.0 (Supplementary Fig. 8c, w=0.01), which is slightly lower than that achieved through optical correction (1.0 to 2.5; Supplementary Fig. 8b). A higher Wiener filter constant (w=0.05) yielded greater SNR gains (1.5-2.5) but at the cost of reduced resolution (Supplementary Fig. 8c). Simulations at SNR levels of 32, 16 and 8 showed consistent improvement ratios, with representative results at SNR 16 presented in Supplementary Fig. 8c.
