# Supplementary material for: Phase-based computational adaptive optics enables artifact-free super-resolution microscopy
Source: Commun Eng. 2026 Mar 9;5:75. doi: 10.1038/s44172-026-00622-7 (PMC13100136; doi:10.1038/s44172-026-00622-7)
Supplement: Supplementary file 2 — Description of Additional Supplementary Files [file 44172_2026_622_MOESM2_ESM.pdf]

Description of Additional Supplementary Files:

File name: Supplementary Movie 1

Description: Side view visualization of a 100-nm fluorescent bead image using wide-field microscopy. Positive and negative spherical aberrations were computationally introduced via phase transfer function-based deconvolution of the 3D image. Normalized variance plots, together with corresponding wavefronts and point spread functions (PSFs), are shown below.
