# Supplementary material for: Phase-based computational adaptive optics enables artifact-free super-resolution microscopy
Source: Commun Eng. 2026 Mar 9;5:75. doi: 10.1038/s44172-026-00622-7 (PMC13100136; doi:10.1038/s44172-026-00622-7)
Supplement: Supplementary file 4 — Reporting Summary [file 44172_2026_622_MOESM4_ESM.pdf]

Corresponding author(s): Atsushi Matsuda

Last updated by author(s): Jan 30, 2026

Reporting Summary

Nature Portfolio wishes to improve the reproducibility of the work that we publish. This form provides structure for consistency and transparency in reporting. For further information on Nature Portfolio policies, see our Editorial Policies and the Editorial Policy Checklist.

Statistics

For all statistical analyses, confirm that the following items are present in the figure legend, table legend, main text, or Methods section.

n/a	Confirmed	
☐	☒	The exact sample size (*n*) for each experimental group/condition, given as a discrete number and unit of measurement
☐	☒	A statement on whether measurements were taken from distinct samples or whether the same sample was measured repeatedly
☒	☐	The statistical test(s) used AND whether they are one- or two-sided<br>*Only common tests should be described solely by name; describe more complex techniques in the Methods section.*
☒	☐	A description of all covariates tested
☒	☐	A description of any assumptions or corrections, such as tests of normality and adjustment for multiple comparisons
☐	☒	A full description of the statistical parameters including central tendency (e.g. means) or other basic estimates (e.g. regression coefficient) AND variation (e.g. standard deviation) or associated estimates of uncertainty (e.g. confidence intervals)
☒	☐	For null hypothesis testing, the test statistic (e.g. *F*, *t*, *r*) with confidence intervals, effect sizes, degrees of freedom and *P* value noted<br>*Give P values as exact values whenever suitable.*
☒	☐	For Bayesian analysis, information on the choice of priors and Markov chain Monte Carlo settings
☒	☐	For hierarchical and complex designs, identification of the appropriate level for tests and full reporting of outcomes
☒	☐	Estimates of effect sizes (e.g. Cohen's *d*, Pearson's *r*), indicating how they were calculated

Our web collection on statistics for biologists contains articles on many of the points above.

Software and code

Policy information about availability of computer code

Data collection	PhiCAO executable is available at https://doi.org/10.5281/zenodo.15042907
Data analysis	Conventional deconvolution was performed by Deconwolf (v0.4.5), IVE Priism suite (v4.2.7), and DeconvolutionLab2 (v2.1.2). Multichannel image alignment and 3D drift correction were performed using Chromagnon (v0.95). Additional codes used for analyses in this study are available upon request.

For manuscripts utilizing custom algorithms or software that are central to the research but not yet described in published literature, software must be made available to editors and reviewers. We strongly encourage code deposition in a community repository (e.g. GitHub). See the Nature Portfolio guidelines for submitting code & software for further information.

Data

Policy information about availability of data

All manuscripts must include a data availability statement. This statement should provide the following information, where applicable:

- Accession codes, unique identifiers, or web links for publicly available datasets
- A description of any restrictions on data availability
- For clinical datasets or third party data, please ensure that the statement adheres to our policy

The image data generated in this study are available in Zenodo with the identifier https://doi.org/10.5281/zenodo.15826325

Research involving human participants, their data, or biological material

Policy information about studies with human participants or human data. See also policy information about sex, gender (identity/presentation), and sexual orientation and race, ethnicity and racism.

Reporting on sex and gender	Not applicable: This study does not involve human participants, their data, or biological material.
Reporting on race, ethnicity, or other socially relevant groupings	N/A
Population characteristics	N/A
Recruitment	N/A
Ethics oversight	N/A

Note that full information on the approval of the study protocol must also be provided in the manuscript.

Field-specific reporting

Please select the one below that is the best fit for your research. If you are not sure, read the appropriate sections before making your selection.

☒ Life sciences ☐ Behavioural & social sciences ☐ Ecological, evolutionary & environmental sciences

For a reference copy of the document with all sections, see nature.com/documents/nr-reporting-summary-flat.pdf

Life sciences study design

All studies must disclose on these points even when the disclosure is negative.

Sample size	For simulation study, >5 replicates were used, for measurements of FWHM, >10 beads were measured.
Data exclusions	No data were excluded from the analysis
Replication	All experiments were repeated more than three times. All attemps at replication were successful.
Randomization	No randomization was performed.
Blinding	No blinding was performed.

Reporting for specific materials, systems and methods

We require information from authors about some types of materials, experimental systems and methods used in many studies. Here, indicate whether each material, system or method listed is relevant to your study. If you are not sure if a list item applies to your research, read the appropriate section before selecting a response.

Materials & experimental systems

n/a	Involved in the study	
☐	☒	Antibodies
☐	☒	Eukaryotic cell lines
☒	☐	Palaeontology and archaeology
☐	☒	Animals and other organisms
☒	☐	Clinical data
☒	☐	Dual use research of concern
☐	☒	Plants

Methods

n/a	Involved in the study	
☒	☐	ChIP-seq
☒	☐	Flow cytometry
☒	☐	MRI-based neuroimaging

Antibodies

Antibodies used	α-tubulin (Woods, A. et al. (1989)), anti-SYP-1, anti-SYP-1(T452p) (Sato-Carlton, A. et al (2017)), Alexa Fluor 488-conjugated secondary antibody (Thermo Fisher Scientific), Dylight 488 and 594 (Jackson ImmunoResearch)

Validation	Primary antibodies were validated in the references, secondary antibodies were all commercially available and validated by manufacturers

Eukaryotic cell lines

Policy information about cell lines and Sex and Gender in Research

Cell line source(s)	HeLa cells were obtained from Riken Cell Bank (RCB007).
Authentication	The cell line was not authenticated.
Mycoplasma contamination	The cell line was not tested for mycoplasma.
Commonly misidentified lines (See ICLAC register)	None.

Animals and other research organisms

Policy information about studies involving animals; ARRIVE guidelines recommended for reporting animal research, and Sex and Gender in Research

Laboratory animals	C. elegans wild-type N2 strain, XA3501(unc-119(ed3) ruls32 III; ojls1), and a strain of genotype mGFP::dsb-1
Wild animals	No wild animals were used.
Reporting on sex	Nematodes are hermaphrodites.
Field-collected samples	The study did not involve field-collected samples.
Ethics oversight	The recombinant DNA experiments conducted in this study were carried out under an appropriate management framework and in compliance with the safety management standards established by the National Institute of Information and Communications Technology (NICT), Kyoto University, the City of Kobe, and the City of Kyoto. Other ethics issues are not applied to this study.

Note that full information on the approval of the study protocol must also be provided in the manuscript.

Plants

Seed stocks	P. patens ssp. patens Gransden strain
Novel plant genotypes	No novel plant genotypes were generated in this study.
Authentication	Plants used in this study were not authenticated.
